# Modeling the Initiation Phase of the Catalytic Cycle in the Glycyl-Radical Enzyme Benzylsuccinate Synthase

**DOI:** 10.1021/acs.jpcb.4c01237

**Published:** 2024-06-07

**Authors:** Maciej Szaleniec, Gabriela Oleksy, Anna Sekuła, Ivana Aleksić, Rafał Pietras, Marcin Sarewicz, Kai Krämer, Antonio J. Pierik, Johann Heider

**Affiliations:** † Jerzy Haber Institute of Catalysis and Surface Chemistry, Polish Academy of Sciences, Kraków 31-201, Poland; ‡ Department of Biology, Laboratory for Microbial Biochemistry, Philipps University Marburg, Marburg 35043, Germany; § Department of Molecular Biophysics, Faculty of Biochemistry, Biophysics and Biotechnology, Jagiellonian University, Kraków 31-007, Poland; ∥ Biochemistry, Faculty of ChemistryRPTU Kaiserslautern-Landau, Kaiserslautern D-67663, Germany; ⊥ Synmikro-Center for Synthetic Microbiology, Philipps University Marburg, Marburg 35043, Germany

## Abstract

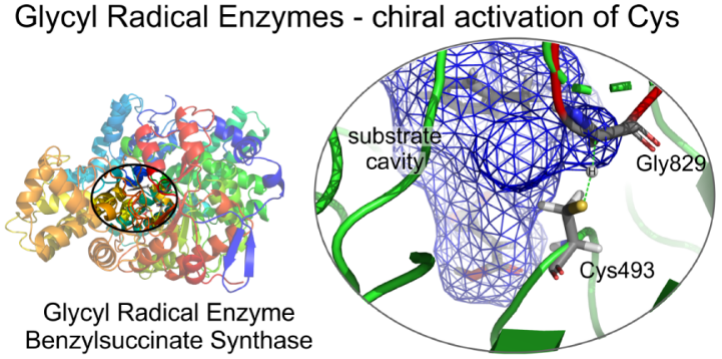

The reaction of benzylsuccinate synthase, the radical-based addition of toluene to a fumarate cosubstrate, is initiated by hydrogen transfer from a conserved cysteine to the nearby glycyl radical in the active center of the enzyme. In this study, we analyze this step by comprehensive computer modeling, predicting (i) the influence of bound substrates or products, (ii) the energy profiles of forward- and backward hydrogen-transfer reactions, (iii) their kinetic constants and potential mechanisms, (iv) enantiospecificity differences, and (v) kinetic isotope effects. Moreover, we support several of the computational predictions experimentally, providing evidence for the predicted H/D-exchange reactions into the product and at the glycyl radical site. Our data indicate that the hydrogen transfer reactions between the active site glycyl and cysteine are principally reversible, but their rates differ strongly depending on their stereochemical orientation, transfer of protium or deuterium, and the presence or absence of substrates or products in the active site. This is particularly evident for the isotope exchange of the remaining protium atom of the glycyl radical to deuterium, which appears dependent on substrate or product binding, explaining why the exchange is observed in some, but not all, glycyl-radical enzymes.

## Introduction

**Figure 1:**
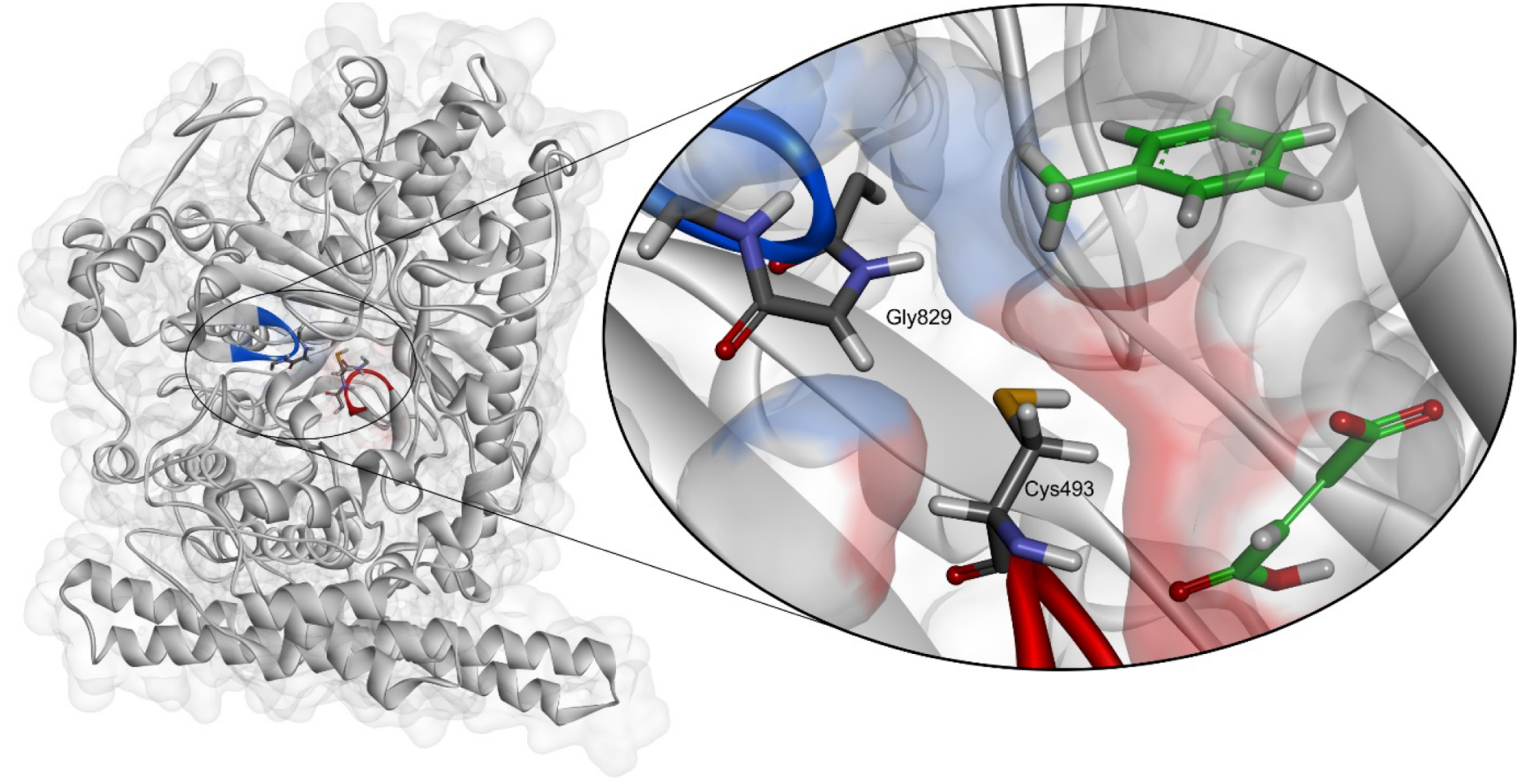
Benzylsuccinate synthase catalytic subunit showing the G- and C-loops carrying radicals Gly829 and Cys493 (blue and red, respectively). The zoomed section indicates details of the active site with bound substrates (green) and the border surface of the active site cavity (semitransparent blue and red color gradient).

Benzylsuccinate synthase^1,2^ belongs to the family of fumarate-adding enzymes (FAE)^3,4^ which is itself part of the growing superfamily of glycyl radical enzymes (GRE).^5−8^ GRE are involved in surprisingly different but always chemically demanding reactions in anaerobic metabolic pathways of bacteria, archaea, and eukarya. In addition to FAE, the currently known families of GRE consist of the pyruvate formate lyases (PFL),^9,10^ type III anaerobic ribonucleotide reductases (ARNR),^11,12^ glycerol^13^ or diol dehydratases^14^ hydroxyproline dehydratases^15^ arylacetate decarboxylases,^16−19^ choline,^20−22^ or isethionate lyases,^23,24^ and phosphonate-cleaving C–P lyases.^25^ They catalyze the formation, cleavage, or rearrangement of C–C, C–O, C–N, C–S, or C–P bonds in biomolecules via radical-based addition or elimination mechanisms.^5,6^ The glycyl-radical-carrying C–P lyases and YfiD-like proteins are excluded from the following overview because the former share no similarity to other GRE^25^ and the latter contain only a small C-terminal domain similar to the glycyl radical site and act as repair system for oxygen-inactivated PFL.^26,27^ The other known GRE contain a homologous large subunit of approximately 100 kDa, which forms a conserved fold, consisting of a 10-stranded βα-barrel with strands of alternating orientations, and two finger loops protruding toward the center and facing each other from opposite sides of the inner wall. Each of these loops carries a strictly conserved amino acid at its tip which are crucial for the reactivity of GRE: a glycine residue located close to the C-terminus and a cysteine at around the middle of the sequences of the subunits^5,6^ (see Figure 1).

All GRE species are initially synthesized in a catalytically inactive state and need to be converted to the active, radical-containing state by a separate activating enzyme, which is a member of the S-adenosylmethionine (SAM)-dependent radical enzymes. The activating enzymes contain a bound SAM cofactor at a special Fe_4_S_4_ cluster in their active centers and form a productive reaction complex with the conserved Gly residues of the corresponding GRE, which are assumed to be pulled out of the folded GRE structures together with their C-terminal ends during activation.^18,28−30^ The activating enzyme then transfers one electron from a low-potential donor like ferredoxin or flavodoxin to the bound SAM cofactor, reducing it to methionine and a Fe-bound deoxyadenosine radical.^31−35^ This is then believed to remove the pro-*S* hydrogen of the conserved Gly residue stereospecifically, based on structural analysis of the PFL-activating enzyme with a bound peptide representing the glycyl radical site^36^ and activation experiments with peptides containing mutations of the glycine to either (*S*)- or (*R*)-alanine. Among these two substrates, only the unnatural (*R*)-alanine carries a pro-*S* hydrogen and is activated, while the conventional peptide is not turned over.^31^ After the glycyl radical is formed, the reduced 5′-deoxyadenosine and the C-terminal peptide of the GRE are released from the activating enzyme, and the GRE refolds to its active, radical-containing state. Because of mesomeric interchange with the electrons of the peptide bond, the glycyl radical is highly stabilized, but only under strictly anaerobic conditions.^34,37,38^ Any contact of an activated GRE with molecular oxygen results in irreversible destruction by oxygenolytic cleavage of the peptide bond at the site of the glycyl radical, as demonstrated for PFL and BSS.^1,39^ This apparently complicated indirect method of initiating a radical reaction is especially advantageous for catabolic reactions occurring multiple times: instead of the high cost of converting one SAM cofactor to methionine and 5′-deoxyadenosine with every reaction of a SAM radical enzyme, this occurs only once per catabolic GRE during its activation. The introduced stable glycyl radical is continuously recycled, allowing a multitude of reactions to proceed without reactivation. The general mechanism of GRE is initiated by substrate binding, which closes the active site and triggers a cascade of hydrogen atom transfer steps, leading to a succession of radical intermediates in the reaction mechanism. Starting with the glycyl radical, a more reactive thiyl radical is generated at the conserved Cys, which then reacts with the bound substrate to create an enzyme-bound substrate radical. To be able to access either the glycyl radical behind the wall of the active site cavity or the bound substrate inside the cavity, the active site Cys (Figure 1, inset) needs to pass back and forth over the cavity wall. The generated substrate radical then undergoes the intended conversion reaction, which is typically not possible in the nonradical state, producing an enzyme-bound product radical species. Finally, the hydrogen atom transfer reactions are repeated in a reverse cascade via the thiyl and glycyl radical intermediates, generating the product in the active site. These reactions are believed to require a tightly closed enzyme–substrate complex to protect the radical intermediates from reacting with interfering molecules from the solvent. Only when the stable glycyl radical state is reached again, the active site can safely be opened to release the product and bind new substrate(s).

While most GRE contain only the conserved Gly and Cys residues as obvious active site elements, some are equipped with additional components required for activity. In particular, the PFL family contains two consecutive conserved Cys in place of just one in all other GRE (Cys418 and 419 in *Escherichia coli*), which leads to an extended hydrogen atom transfer cascade with two thiyl intermediates instead of the single one involved in other GRE. Although several versions of the reaction mechanism have been proposed over time,^40−42^ it is clear that both Cys are essential for the mechanism, and that Cys418 is involved in covalent binding of a substrate radical derived from pyruvate.^40,43^ In contrast to initial proposals favoring thiyl radical formation at Cys418 by direct interaction with the glycyl radical,^40,42^ later studies suggest that the initial thiyl radical is formed at Cys419.^44,45^ Instead of directly binding pyruvate to the thiyl of Cys419 and transferring the subsequently formed acetyl-thioester to Cys418, as considered earlier,^42,44^ the Cys419 thiyl moiety is currently considered to act as “radical hub” to form a thiyl on Cys418, which then forms a covalent bond with the carbonyl carbon of pyruvate.^41^ This intermediate is then homolytically cleaved to a formyl radical and an acetyl thioester at Cys418. The latter is transferred to CoA, while the released formyl radical thiyl restores the thiyl radical at Cys419.^40,41,46−48^ A second exception is known in ARNR that contains a structural Zn-binding site in their glycyl radical domain, whose function is still unclear. In addition, the ARNR need further compounds inside the active site to serve as suitable reductants because the reaction requires an additional two-step electrochemical reduction step after the initial radical-dependent dehydration of the ribose of a bound ribonucleotide triphosphate to a 2′-deoxy-3′-keto product radical species.^6,49^ In recent years, it became clear that at least three different subtypes of the type III-ARNR exist which differ in the required reductant and the presence of conserved amino acids involved in mechanistic details.^6^ The best-characterized subtype is represented by ARNR of *E. coli*, which accommodates an additional formate molecule in the active site that serves in the reaction mechanism simultaneously as acid and reductant.^6,11,49,50^ The other two subtypes of ARNR have been identified in *Neisseria bacilliformis* and *Methanosarcina barkeri*, which use thioredoxin or a glutaredoxin-like protein as reductant instead of formate.^51,52^ Finally, the enzymes of FAE and aromatic acid decarboxylase families contain additional subunits carrying FeS-clusters. In the case of BSS, two small subunits with one Fe_4_S_4_ cluster each have been observed,^1,53,54^ whereas 4-hydroxyphenylacetate decarboxylase contains one small subunit with two Fe_4_S_4_ clusters.^55,56^ In either case, the functions of these subunits are still unclear.

The glycyl radical has been characterized by EPR spectroscopy in many of these enzymes, and they show remarkably similar spectroscopic features (Table 1). The remaining H atom at C_α_ of the glycyl radical residue causes a characteristic pronounced hyperfine splitting of 1.5 mT (1.7 mT for radiation-induced glycyl radicals in small model substrates^63^) in the observed X-band EPR spectra. In PFL and BSS, this hydrogen atom has been reported to be rapidly exchanged to deuterium in D_2_O-based solvents, while such an exchange did not occur in ARNR.^59,60^ For PFL, only Cys419, but not Cys418 of the conserved Cys pair, was shown to be involved in the observed H/D isotope exchange of the glycyl radical by a mutagenesis study.^44^ However, the detailed mechanism of this process is still unknown for any GRE.

**Table 1:** *g*-values of the Glycyl Radical Signals in Various GRE^a^

enzyme	family	*g*_av_	*g*_x_	*g*_y_	*g*_z_	H/D exchange	reference
pyruvate formate lyase	PFL	2.0037	2.0047	2.0039	2.0025	yes	(39,58)
anaerobic ribonucleotide reductase	ARNR	2.0033	2.0043	2.0033	2.0023	no	(58−61)
benzylsuccinate synthase	BSS	2.0034*	2.0045	2.0036	2.0022	yes	(57,58,62)
N-acetylglycyl radical		2.0032	2.0045	2.0031	2.0020	no	(58,63)

a The values measured in X-band experiments are given as *g*_av_, and those under very-high-field conditions (525 GHz microwave frequency at up to 20 T magnetic flux density) as *g*_x,y,z_. An organic glycyl radical model is added for reference. *A deviating *g*_av_ value of 2.0021 was initially reported^57^, which may be due to improper calibration.

In this study, we report on computational modeling of the radical transfer steps between the active site Gly and Cys that are involved in activating the bound substrate, using BSS as a model system with bound substrates or products and the apoenzyme as a reference. From these models, we infer a hypothesis as to why isotope exchange of the hydrogen atom of the glycyl radical site has been observed in some but not in all GRE.

## Materials and Methods

### Bacteria Cultivation

For the cultivation of *Aromatoleum sp*., minimal medium (“*Thauera* broth” Thb) was used,^64^ with some minor modifications. For the preparation of this medium, the main solution was made anaerobically and autoclaved separately, containing 816 mg/L KH_2_PO_4_, 5920 mg/L K_2_HPO_4_, 530 mg/L NH_4_Cl, 200 mg/L MgSO_4_, 1000 mg/L KNO_3_, and 25 mg/L CaCl_2_ x H_2_O. After autoclaving, the medium was supplemented with 10 mL/L trace elements, 5 mL/L vitamins solution, and 1 mL/L sodium selenite/sodium tungstate stock solution; stock solutions were (1000x). The precultures of *Aromatoleum sp*. were prepared in Thb medium supplemented with 4 mM sodium benzoate, allowing the bacteria to reach the stationary stage before inoculating it into a medium with 0.025% toluene in a larger volume (1–2 L). To prevent toluene toxicity, paraffin oil was added to the medium at a final concentration of 2%. Further on, the culture was supplemented with 0.1% toluene in the final volume when needed, based on monitoring nitrate and nitrite levels, assuming a 1:4 ratio of toluene and nitrate consumption. The bacteria were grown anaerobically at 28 °C on a horizontal platform under agitation at 180 rpm. The incubation period was 14 days or until the culture reached an optical density (OD_600 nm_) of at least 2–2.5.

### Preparation of Cell Extract

Fully grown cultures were opened under anaerobic conditions (97:3 N_2_:H_2_) and moved into centrifugation beakers. Cells were harvested at 4500 x *g* and 4 °C for 45 min. The cell pellet was resuspended in a 1:1 ratio with 20 mM triethanolamine (TEA)/HCl buffer pH 7.8 and homogenized by ultrasonication (20 mHz, 20% amplitude, 3 s pulse with 9 s pause for 20 min; Sonifier 250, Branson) while cooled with solid ice packs to prevent denaturation. Lysed cells were ultracentrifuged at 100 000 x *g*, 4 °C for 1 h. The supernatant was collected, and the obtained cell-free extract was stored at −80 °C until use under a blanketing atmosphere of N_2_/H_2_. The protein concentration of the cell extract was determined in a 2 μL spectroscopic method (260 and 280 nm, BioTek with Take3 plate) yielding 39 ± 0.9 mg/mL.

### Activity Assay with Toluene

The activity of BSS was measured at 30 °C in a thermoblock according to the following procedure: 0.2 mL of cell-free extract (39 mg/mL) was suspended in 0.73 mL of 20 mM TEA/HCl buffer, pH 7.8, to which 50 μL of 100 mM sodium fumarate in water was added, yielding a final concentration of 5 mM. After 2 min of incubation, reactions were started by the addition of 8.2 μL toluene dissolved in isopropanol (stock concentration of 364.5 mM, established by UV–vis at 260 nm, ε = 223 ± 3 M^–1^ cm^–1^) to the final concentration of 3 mM. The isopropanol content in each reaction mix was adjusted to 2%. 150 μL of samples were collected at 0, 5, 10, 15, and 20 min, mixed in a 1:1 ratio with acetonitrile and centrifuged (8000 x *g*, 20 min) to remove protein residues. The supernatants of the 1:1 diluted assays were directly analyzed by LC-DAD, while samples for LC-MS/MS analysis were diluted 30-fold with acetonitrile. All experiments were carried out in triplicates. The usual activity measured with toluene in the extract was in the range of 5.6 nmol min^–1^ [mg of protein in cell extract]^−1^.

### H/D Exchange in Benzylsuccinate in D_2_O

The reaction mixture comprised 0.2 mL of cell-free extract, 0.4 mL of 20 mM TEA/HCl buffer pH 7.8, and 0.4 mL of D_2_O (Sigma-Aldrich 99.9%). The mixture was incubated with *rac*-benzylsuccinate at the concentration of approximately 10 mg/L (48 μM) at 30 °C which was enriched by (*R*)-benzylsuccinate present in the cell extract (3–4 μM). As control reactions, we conducted the assays with added (*S*)-benzylsuccinate (48 μM) or oxygen-inactivated enzyme and *rac*-benzylsuccinate. 150 μL samples were collected from each reaction vessel at times 0, 10, 20, 30, and 240 min, mixed in a 1:1 ratio with acetonitrile (LC-MS grade), and centrifuged. Supernatants were diluted 5 times with acetonitrile and subjected to LC-MS analysis. Reactions were carried out in triplicates.

### D/H Exchange during Reaction with d_8_-Toluene and Fumarate in H_2_O

To the reaction mixture comprised of 0.2 mL of cell-free extract, 0.73 mL of 20 mM TEA/HCl buffer pH 7.8, 5 mM sodium fumarate was added from a 100 mM stock solution in water. After 2 min of incubation at 30 °C in a thermoblock, reactions were started by adding 8.4 μL d_8_-toluene dissolved in isopropanol (stock concentration of 358.2 mM) to a final concentration of 3 mM. The isopropanol content in each reaction mix was adjusted to 2%. Samples of 150 μL were collected at 0, 5, 10, 15, and 20 min, and the reaction was stopped by mixing these in a 1:1 ratio with acetonitrile. These diluted supernatants were processed as described above and analyzed with LC-DAD, while samples to be analyzed by LC-MS/MS were 20x diluted with acetonitrile. All experiments were carried out in triplicates.

### UHPLC-DAD-MS/MS

Samples were analyzed on an Agilent 1260 UHPLC instrument coupled with DAD and an Agilent 6460 Triple Quad MS detector. Analytes were injected in a volume of 2 μL on a ZORBAX 300 SB-C18 column (RRHD, 2.1 × 50 mm, 1.8 μm, Agilent) and eluted at a flow rate of 0.4 mL/min by a gradient method, employing Millipore deionized water (A) and acetonitrile (B), acidified with 0.01% HCOOH. The gradient program was as follows: 0–1.0 min 15% B, 1.0–3.5 min 45% B, 3.5–4.0 min 75% B, 4.0–5.0 min 15% B. Column temperature was thermostated at 30 °C. The peak of benzylsuccinate eluated at 2.3 min.

The quantitative analysis of benzylsuccinate was conducted using a DAD detector at 210 nm and external calibration (Figure S1A) for samples diluted 1:1 with acetonitrile. This method was used for the determination of the enzyme activity with toluene and d_8_-toluene.

The H/D exchange process was analyzed with electrospray ionization MS with a negative polarization (see Tables S1–S4 for parameters). H/D exchange in a product of enzymatic reaction with d_8_-toluene was examined by following the characteristic peaks of [M-H]^−^ quasi-molecular ions of d_8_-benzylsuccinate and d_7_-benzylsuccinate (215 and 214 *m*/*z*, respectively) in MRM mode (following fragmentation ions of 171 or 170 *m*/*z*). The signal ratio from MS was combined with quantitative analysis from DAD to obtain concentrations of analytes.

H/D exchange in benzylsuccinate in D_2_O was monitored by single ion monitoring mode (SIM2), following the signals of 207 *m*/*z* (corresponding to quasi-molecular ion of benzylsuccinate [M-H]^−^), 208 *m*/*z* (corresponding to d_1_-benzylsuccinate [M-H]^−^ peak and [M+1-H]^−^ of benzylsuccinate), and 209 *m*/*z* (corresponding to [M+2]^−^ of benzylsuccinate, [M+1-H]^−^ of d_1_-benzylsuccinate and [M-H]^−^ of d_2_-benzylsuccinate) using calibration with external standard (Figure S1B).

The fragmentation patterns of benzylsuccinate and its labeled derivatives were analyzed in product ion mode (MS parameters, Tables S1–S4). All samples were analyzed in duplicate.

### H/D Exchange for EPR

Samples for the EPR monitoring of H/D exchange on the glycyl radical were prepared by exchanging the solvent via passage over PD-10 gel filtration columns (1.5 cm × 5 cm, GE HealthCare). These were made anoxic by washing with water containing 5 mM dithionite, then equilibrated with either anoxic water or D_2_O. After applying 0.5 mL of cell extract, the columns were eluted with either anoxic water or D_2_O, resulting in an initial exchange of water to D_2_O and a second exchange back to water. The recovered protein-containing eluates did not show a significant dilution. The exchange of protium to deuterium was also confirmed by mixing the cell-free extract in a 1:1 ratio with anoxic D_2_O, using nontreated cell extract as a control sample. Extracts were transferred into EPR tubes under anaerobic conditions, secured with a clamped rubber tube, and gently frozen in liquid nitrogen. The samples were stored in liquid nitrogen until measured.

### EPR Measurements

The EPR spectra for H/D exchange of the glycyl radical and the reverse exchange were observed in cell extracts of toluene-grown *Aromatoleum toluolicum* strain T. They were recorded with an EMX-6/1 X-band spectrometer (Bruker, Karlsruhe, Germany) with a standard TE102 rectangular cavity and an ESR-900 helium flow cryostat with variable temperature (Oxford instruments, Oxford, UK) or a liquid-nitrogen finger dewar as described in (62). The H/D exchange at the glycyl radical in D_2_O was also confirmed by EPR spectra of extracts of toluene-grown cells of an *Aromatoleum sp*., which were recorded by a Bruker Elexsys E580 spectrometer and SHQ4122 resonator equipped with an ESR900 cryostat (Oxford Instruments) (see Supporting Information for details).

### BSS Model Preparation

The initial structure of the α subunit of BSS T1 in complex with monoprotonated fumarate and toluene was obtained from the crystal structure (PDB codes: 5BWD, 5BWE)^65^ as described previously by Salii et al.^66^

The initial structure of the BSS T1 apoenzyme was obtained from the crystal structure of the apoenzyme (PDB code: 4PKF).^54^ The water molecules as well as β and γ subunits were removed, and the model was protonated with H++ at pH 7.4.^67^ The overall charge of the model was −3 which was equilibrated with sodium ions.

The AMBER parameters for radical Gly829 were taken from Barone et al.^68^ The BSS models were solvated with explicit water molecules (10 Å radius around the protein 23 324 or 24 215 water molecules, respectively for holo- and apoenzyme), and the calculations were conducted in a periodic-boundaries box (119.6 Å × 86.3 Å × 96.8 Å). The AMBER parameters for radical Cys493 were derived according to standard protocols.^69^ All AMBER parameters of enzyme ligands and nonstandard residues are provided in Supporting Information.

### MD Simulation

All classical MD simulations were performed for holo and apo BSS models using the AMBER ff03 force field.^70^ The calculations were conducted with AMBER 18^71^ according to the previously described protocol.^71^ The stable parts (i.e., final 20 ns) of the 50–60 ns simulation trajectories, i.e., exhibiting stable RMSD of the main chain (Figure S4), were analyzed with clustering using the k-means method taking into consideration heavy atoms of the active site residues (see Supporting Information).

For the apoenzyme, the optimal number of 6 clusters was selected based on the DBI and pSF indexes. A structure for further QM:MM calculations was selected based on the following criteria: the size of the cluster (the higher the better), cluster tightness (the average distance from the centroid, the lower the better), silhouette (the higher the better), and the agreement of the Cys493 C–Cα-Cβ-Sγ dihedral angle with its median value observed during MD simulation.

For the enzyme–substrate complex, we analyzed the last 35 ns of the stable trajectory using the same approach. However, in this case, clustering yielded geometries with toluene at the entrance of the active site, far from Cys493. Therefore, for QM:MM calculations, we selected the frame with the shortest distance (3.23 Å) of the toluene group to the SH group of Cys493, which belongs to the first cluster which described the majority (54%) of the analyzed frames. The selected geometries were minimized prior to the preparation of the QM:MM models using the 3-stage protocol (see Supporting Information). However, the statistical parameters for E:S were derived from 4 simulations of 62 ns (Figure S5).

For simulations of the enzyme with radical Cys493, we conducted three 60 ns simulations with toluene and monoprotonated fumarate as well as with monoprotonated (*R*)-benzylsuccinate, while for apoenzymes, two 100 ns simulations were used (Figure S6). For the sections of trajectories with stable RMSD, we analyzed the distances between Cα atoms of Gly928 and Cys493 (Figure S7).

### QM:MM Modeling

**Figure 2:**
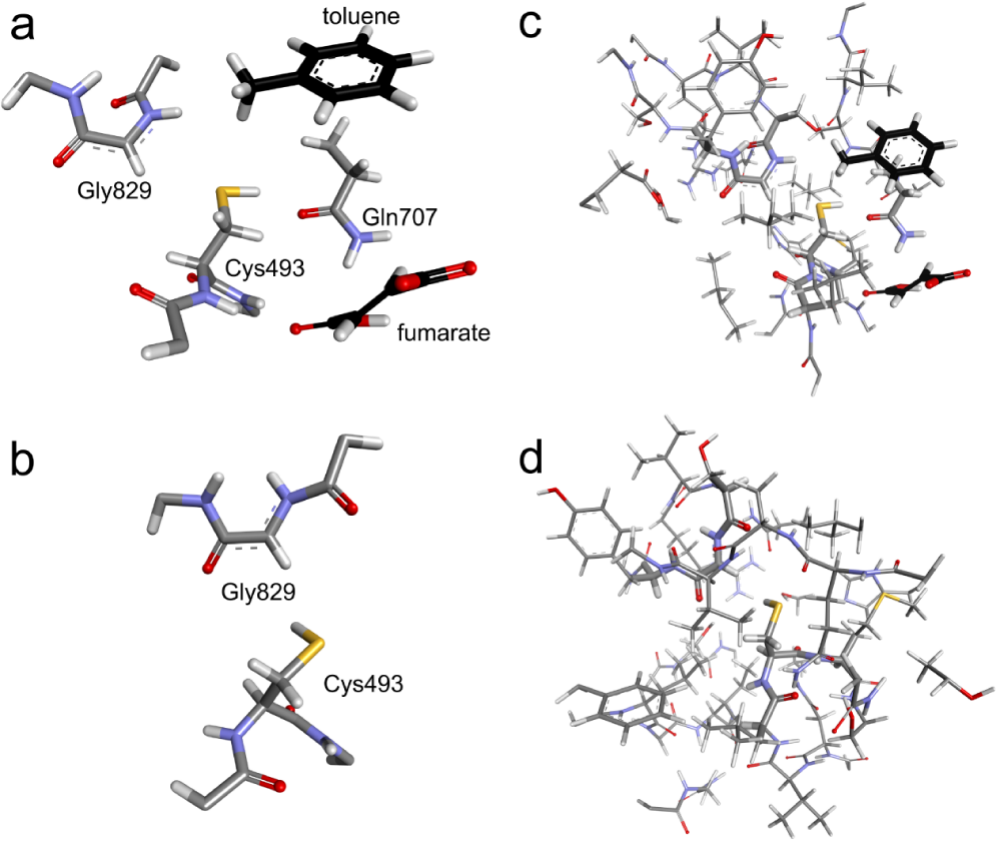
QM (HL) part of the QM:MM BSS models. S-HL for a) holoenzyme and b) apoenzyme models; B-HL for c) holoenzyme and d) apoenzyme models.

All QM:MM calculations were conducted using the Gaussian16 C.01 program.^72^ The QM:MM models obtained from MD simulations were stripped from sodium ions and most of the water molecules, leaving only H_2_O molecules penetrating a 20 Å radius from Cys493. The positions of all residues and water molecules above a 15 Å radius from Cys493 were frozen in geometry optimization. The overall charges of the QM:MM models were −7 and −3 for holo- and apo-BSS models, respectively. The fumarate was modeled in the monoprotonated state according to previous docking studies.^73^ Two sizes of high layer (HL) were used in the study: a small one (S-HL, Figure 2a,b) used for geometry optimization and vibrational analysis and a big one (B-HL, Figure 2c,d) used for single point correction of the energy.

The S-HL of the apoenzyme model was composed of Cys493 and Gly829 residues with adjacent fragments of the main chain (Figure 2b). The S-HL of the holoenzyme model (Figure 2a) was extended by the residue of Gln707 as well as fumarate and toluene with respect to that of the apoenzyme. The B-HL comprised of S-HL extended by all residues penetrating a 5 Å radius of Gly829 and Cys493 for the apoenzyme (389 atoms), and a 5 Å radius of Gly829 with a 3 Å radius of Cys493 plus toluene and fumarate for the holoenzyme (315 atoms). The charges of the S-HL were 0 and −1, and those for the B-HL were 1 and 0, respectively. All calculations were conducted for a doublet state due to the presence of a single radical.

The geometry of the QM:MM models was optimized at the B3LYP/6-31g(d,p):AMBER level of theory using an electronic embedding approach,^74^ which was followed by vibrational analysis introducing vibrational corrections for stationary points. The transition states of the H atom transfer between Cys493 and Gly829 were localized by means of relaxed scans along the reaction coordinate (i.e., d(Cys–S-H^···^C^rad^-Gly)) followed by TS optimization using the Berny algorithm. Each stationary state preceding or following a particular TS was optimized individually, with initial geometry derived from the TS. The energy of the final stationary points was corrected with single point calculations using B-HL at B3LYP/6-311g+(2d,2p):AMBER level of theory with electronic embedding and Grimme D3 corrections for disperse interactions.^75^ The only exception from this protocol was the pro*S* intermediate of the apoenzyme (pro*S* I^apo^) for which we were able to calculate energy only at the B3LYP/6-31g(d,p):AMBER level of theory. As a result, the energy for pro*S* I^apo^ was approximated based on the energy difference between pro*S* I^apo^ and pro*S* TS^apo^ calculated at the B-HL/B3LYP/6-31g(d,p)/D3:AMBER level of theory. The vibrational corrections were calculated at 303 K and 1 atm. and scaled with 0.9806 factor according to B3LYP/6-31g(d) correction calculated by Scott and Radom,^76^ and thermal energy correction was added to all electronic energies calculated at the respective level of theory. The corrections for the transfer of the H/D atom were obtained by substitution of the mass of respective protium nuclei with an isovalue of 2, taking into consideration substitution of the transferred hydrogen atom, atom position at Gly829, both of the hydrogen atoms, or none.

The transfer of the H atom from Cys to Gly was evaluated for the holoenzyme in either substrate (toluene and fumarate) or product ((*R*)-benzylsuccinate) bound state. As a reference, the apoenzyme was examined. For each transition, two alternate conformations were evaluated, leading to H atom transfer toward the *re* or *si* face of radical Gly.

Finally, we have evaluated a potential alternate activation pathway that may proceed via a water molecule instead of the direct H transfer between Cys493 and Gly829. To investigate this, the H_2_O closest to the glycyl radical was included in the S-HL and B-HL of the apo model (see Figure S2). Both sequential and concerted mechanisms were investigated, that either assumed a radical OH intermediate or concerted transfer of a H atom from H_2_O to the glycyl radical and from the cysteine SH group to H_2_O without formation of the OH radical species.

### Kinetic Rate Estimations

The estimation of elementary rate constants used for approximation of the kinetic rate of H/D exchange at Gly829 based on the energy barriers calculated for B-HL at B3LYP/6-311g+(2d,2p)/D3:AMBER level of theory corrected by thermal energy corrections according to a standard equation from transition state theory:
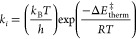
where *k*_B_ is Boltzmann constant, *h* is Planck constant, *R* is gas constant, *T* is 303 K, and the transmission coefficient is assumed to be a unity.

**Figure 3:**
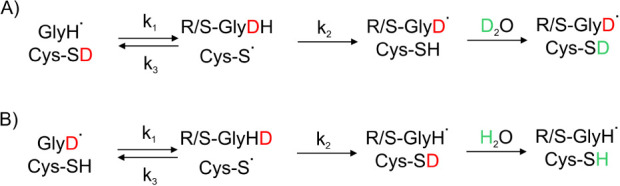
Scheme depicting A) deuteration of the BSS glycyl radical in D_2_O and B) protonation of the deuterated BSS glycyl radical in H_2_O.

The steady-state kinetic constant of the deuteration process (Figure 3A) was described by a standard scheme assuming a two-step reaction with the first reversible step and the second irreversible step. The second step is assumed irreversible due to the H/D exchange of the proton/deuteron with abundant solvent.

For such assumptions, an observed overall kinetic constant of the H/D exchange was calculated according to ref (77) as
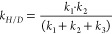


The analogical analysis was also conducted for the process of protonation of the deuterated enzyme, which was introduced to H_2_O (Figure 3B).

## Results

### The Mechanism of Cys493 Activation

The C–C bond formation between fumarate and toluene catalyzed by benzylsuccinate synthase is enabled by the interaction of a thiyl radical of Cys493 with the methyl group of the alkylaromatic substrate, which has been taken as starting point for previous studies on the reaction mechanism.^73^ We have modeled fumarate in a monoprotonated state into the active site based on docking studies favoring a deprotonated charged carboxy group interacting with Arg508, but a protonated one at the second docking site, which consists of protein backbone contacts to Cys493 and neighboring residues.^78^ Analysis of the charge distribution of the latter binding site shows the presence of a negatively charged patch in an otherwise positively charged pocket, which provides a potential target for binding the carboxylic −OH group via an H-bridge and supports the proposed binding of a nondissociated carboxy group (Figure S3).

**Figure 4:**
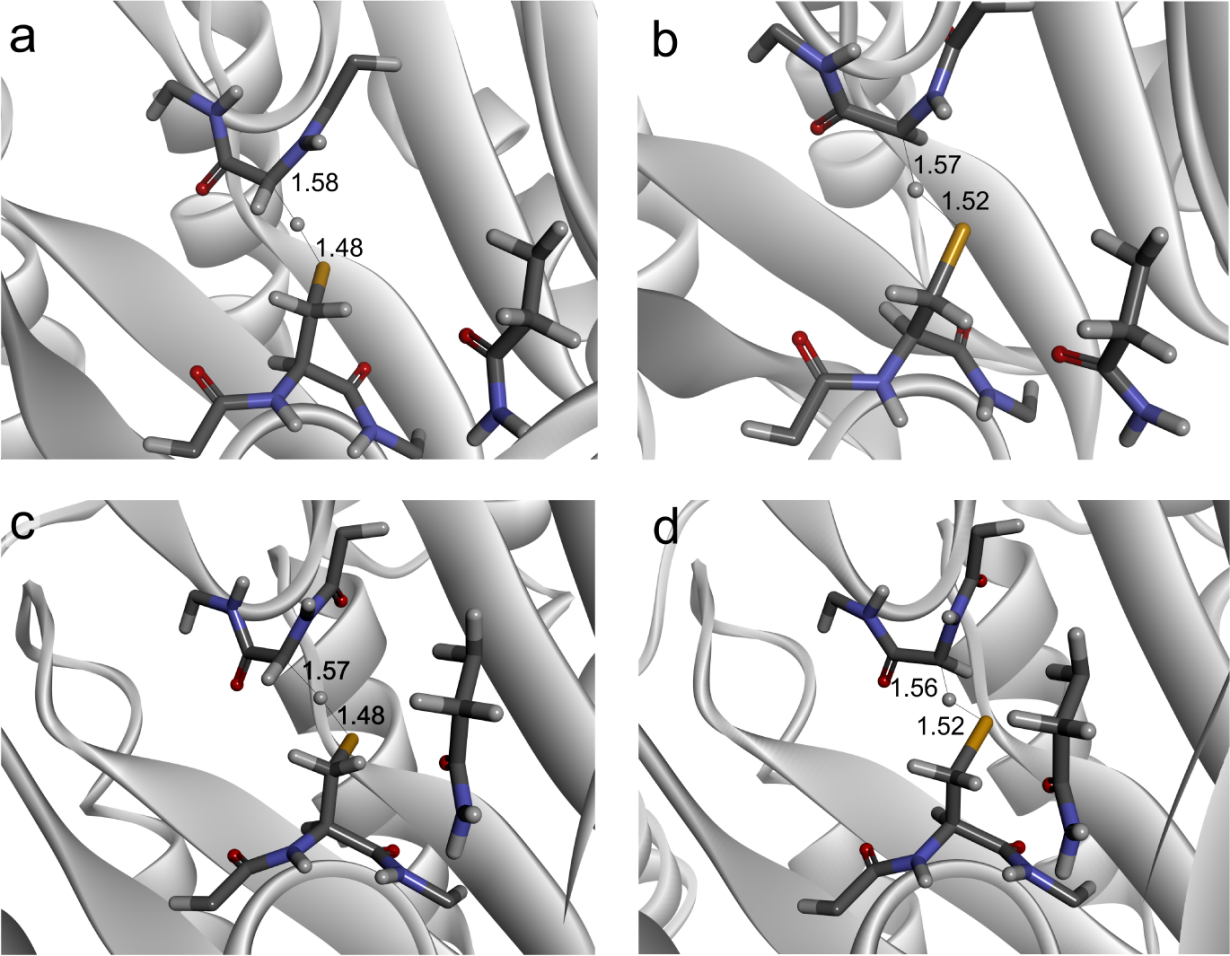
Structure of holoenzyme a) pro*R* TS^ES^ and b) pro*S* TS^ES^ conformation, c) pro*R* TS^EP^ and d) pro*S* TS^EP^. The numbers provide distances in Å. Substrates and products were hidden for clarity.

The reactive thiyl radical, which was used as starting point of our previous mechanistic model,^78^ must first be generated by transferring an H atom from the sulfhydryl group of Cys493 to the radical Gly829.^3^ This crucial initial process occurs in all GRE, but it is poorly understood to date, and almost no approaches have been addressed to obtain computational models. Therefore, we decided to employ QM:MM modeling of this step using BSS as an example. We started our investigation with an enzyme–substrate complex (E:S), where both fumarate and toluene are bound in the active site. The reaction starts with radical Gly829 and the sulfhydryl group of Cys493, which is turned away in the direction of neighboring residue Gln707. The spin density of the initial glycyl radical is mostly located at the Cα carbon of Gly (0.9) but is also distributed by resonance along the peptide backbone to the adjacent nitrogen (0.075) and carbonyl oxygen atoms (0.1) of the preceding peptide bond and with a resulting negative spin density to the carbonyl C atom of Gly829 (−0.16). These values are remarkably similar to previously calculated spin densities of glycyl radicals in crystals of the model compound N-acetylglycine, where the Cα carbon was attributed to a value of 0.77 of spin density.^63^ Both residues, Gly829 and Cys493, represent the tips of two turns, which extend as loops from the inner rim to the center of the barrel structure (hereafter called the G-loop and C-loop). Both residues are essential contributors to the active site of the enzyme and directly face each other.^54^ To transfer the H atom, Cys493 has first to change its conformation, i.e., turn the SH group to face toward the glycyl radical and slightly rotate around the Cα-Cβ bond. Furthermore, a slight bending of the end of the C-loop toward the G-loop shortens the distance between the Cα of the glycyl radical and the SH group of Cys493 from approximately 3.8 to 3.0 Å. From this conformation, two potential types of transition states can occur — the H atom from the SH group can be delivered to either the *re* (pro*R*) or *si* (pro*S*) face of the radical at Gly829. The pro*R*-TS^ES^ involved in hydrogen transfer via the *re* face is facing the side chain of Gln707 and does not require a significant conformational shift of the G-loop. The calculated C–H and S–H distances are 1.58 and 1.48 Å, and the S–H–C angle is almost linear at 167.4° (Figures 4a and S8). The spin density in this TS is divided between the Cα atom of Gly829 (0.61) and the S atom of Cys493 (0.3), with the rest distributed in resonance at the adjacent atoms of Gly829.

A similar transfer can also occur when the H atom is transferred to the *si* face of the glycyl radical (leading to the pro*S*-TS^ES^). The position of the Cys, in this case, resembles that in the pro*R*-TS^ES^, but the G-loop harboring the glycyl radical is bent to present the other face of the Gly. The C–H and S–H distances at pro*S*-TS^ES^ are still quite short at 1.57 and 1.52 Å, while the S–H–C angle is more bent at 145° (Figures 4b and S8). Moreover, the radical density is more localized in pro*S*-TS^ES^ compared to that in pro*R*-TS^ES^, as the spin densities are 0.36 at the S atom and 0.66 at Cα of Gly829.

The H transfer is completed with the formation of the nonradical Gly829 and the thiyl radical at Cys493, which slightly rotates back toward Gln707. The main difference in the structures of both intermediates (I1) is associated with the position of Arg826, which gets into close contact with the G-loop in the pro*S* conformation due to its conformational change.

The analysis of energy barriers calculated for both models by using B-QM, corrected with D3 dispersion and thermal energy corrections (Table 2; see Tables S6 and S7 for all energies), indicates that the pro*R*-TS^ES^ is energetically more favorable (40.4 kJ/mol) and hence more probable compared to the pro*S*-TS^ES^ (62.2 kJ/mol). However, the difference is not large enough to exclude a reaction via pro*S*-TS^ES^, rendering both processes kinetically possible. The energy state of intermediate I1 is very similar in both models and turned out to be 32–34 kJ/mol lower (more stable) than that of the substrate-bound E:S state.

**Table 2:** Energy Barriers Calculated for H Atom Transfer between Cys493 and Gly829 in the Holoenzyme (E:S or E:P) with Either Bound Substrates (TS^ES^) or Product (TS^EP^) or in the Apoenzyme with an Empty Active Site (apo)^a^

Attack		H transfer from Cys to Gly^rad^Δ(E+thermal) [kJ/mol]				Attack		H transfer from Gly to Cys^rad^Δ (E+Thermal) [kJ/mol]			
		*re* H GlyH^•^	*re* D GlyH^•^	*re* H GlyD^•^	*re* D GlyD^•^			*R***-**H GlyH_2_	*R*-D *R*-GlyHD	*R*-H *S*-GlyDH	*R***-**D GlyD_2_
E:S *re*	E:S	0.0	0.0	0.0	0.0	E:S *R*-trans.	I^ES^	0.0	0.0	0.0	0.0
	TS^ES^	40.4	43.2	39.8	42.6		TS^ES^	72.2	77.4	72.4	77.7
	I^ES^	–31.7	–34.2	–32.6	–35.2		E:S	31.7	34.2	32.6	35.2
E:P *re*	E:P	0.0	0.0	0.0	0.0	E:P *R*-trans.	I^EP^	0.0	0.0	0.0	0.0
	TS^EP^	38.8	41.5	38.1	40.8		TS^EP^	72.5	77.7	72.7	78.0
	I^EP^	–33.7	–36.2	–34.6	–37.2		E:P	33.7	36.2	34.6	37.2
apo *re*	E^apo^	0	0	0	0	apo *R*-trans.	I^apo^	0	0	0	0
	TS^apo^	107.0	109.8	106.2	109.0		TS^apo^	99.8	105.0	99.7	104.9
	I^apo^	7.2	4.8	6.5	4.0		E^apo^	–7.2	–4.8	–6.5	–4.0
		*si* H Gly H^•^	*si* D GlyH^•^	*si* H GlyD^•^	*si* D GlyD^•^			*S***-**H GlyH_2_	*S*-D *S*-GlyDH	*S***-**H *R***-**GlyHD	*S***-**D GlyD_2_
E:S *si*	E:S	0.0	0.0	0.0	0.0	E:S *S*-trans.	I^ES^	0.0	0.0	0.0	0.0
	TS^ES^	62.2	64.8	61.5	64.2		TS^ES^	95.8	101.0	96.1	101.4
	I^ES^	–33.7	–36.2	–34.6	–37.2		E:S	33.7	36.2	34.6	37.2
E:P *si*	E:P	0.0	0.0	0.0	0.0	E:P *S*-trans.	I^EP^	0.0	0.0	0.0	0.0
	TS^EP^	61.9	67.8	61.2	67.1		TS^EP^	96.4	104.7	96.6	105.0
	I^EP^	–34.4	–36.9	–35.4	–37.9		E:P	34.4	36.9	35.4	37.9
apo *si*	E^apo^	0	0	0	0	apo *S*-trans.	I^apo^	0	0	0	0
	TS^apo^	80.6	80.1	83.1	82.7		TS^apo^	2.3	2.4	7.4	7.6
	I^apo^	78.3	77.8	75.7	75.1		E^apo^	–78.3	–77.8	–75.7	–75.1

a The electronic energies were corrected with thermal energy calculated for models with protium-only substituted Gly (*re*-/*si*-H GlyH), with either enantiomer of monodeuterated Gly assuming transfer of either its deuterium (*re*/*si*-D GlyH^•^) or protium-substituent (re/si-H GlyD^•^), and with deuterium substituting both H atoms of Gly (*re*/*si* D GlyD^•^). For readers’ convenience, the barriers associated with the respective reverse processes, i.e., transfer of H/D atoms from Gly to radical Cys are provided in the right column (*R*/*S*-H or D transfers). Note that the *R*- and *S*-enantiomers of monodeutered Gly are also represented as GlyHD and GlyDH, respectively, to aid the comprehension of Figure 6.

Assuming a partial reversibility of the BSS reaction between the product and the product radical, we have investigated the same process for the enzyme:product model (E:P) by modeling the steps of the BSS mechanism in reverse. Thus, the process starts with the bound product in the E:P complex, Gly829 in the radical state (spin density at Cα 0.91), and Cys493 in the sulfhydryl form with an SH group. The Cys residue of the E:P complex is localized between the G-loop hosting Gly829 and a tightly packed arrangement of the side chains of residues Met494, Leu766, and Gln707 (Figure S9). The H transfer occurs in analogy to the E:S complex, involving an attack on either the *si* (pro*S*) or *re* (pro*R*) face of the glycyl radical. As expected, the calculated geometries of the transition states are almost identical to those observed for E:S with d(C–H) = 1.57 Å, d(S–H) = 1.48 Å, and S–H–C angle 166.5° for the pro*R* and d(C–H) = 1.56 Å, d(S–H)=1.52 Å, and S–H–C angle 146.2° for the pro*S* conformation (Figure 4c,d). The spin density in pro*R*-TS^EP^ is divided between the Cα atom of Gly829 and the S atom of Cys493 for both the pro*R*- (0.61 and 0.28, respectively) and the pro*S*-directed reactions (0.68 and 0.32, respectively). The geometric and electronic similarities to the E:S complexes correspond also with the very similar calculated values of the energy barriers between E:P and the respective TS^EP^, which were 38.8 kJ/mol for pro*R*-TS^EP^ and 61.9 kJ/mol for pro*S*-TS^EP^. The similarity even extended to the prediction that the first intermediate containing the thiyl radical of Cys493 is energetically more stable (by 33 kJ/mol) than the E:P state with the glycyl radical (Table 2).

Finally, we have also analyzed the same process for the apoenzyme without substrates or product present in the active site. Such a model represents the situation when water molecules can penetrate the active site despite its hydrophobicity and the geometry of the enzyme is not influenced by the strong binding of fumarate. Besides the presence of three water molecules in the active site, the apoenzyme differs slightly in the positions of the C- and G-loops. The model representative for the most populated cluster in statistical analysis of MD trajectories exhibited the tips of the β-turns at a Cα–Cα distance of approximately 5.8 Å, compared to 5.0 Å in the substrate- or product-bound models. In both cases, the overall distributions of the Cα–Cα distances from statistical analysis were in the range of 3.7 and 8.2 Å (medians 5.46 vs 5.28 Å, respectively).

**Figure 5:**
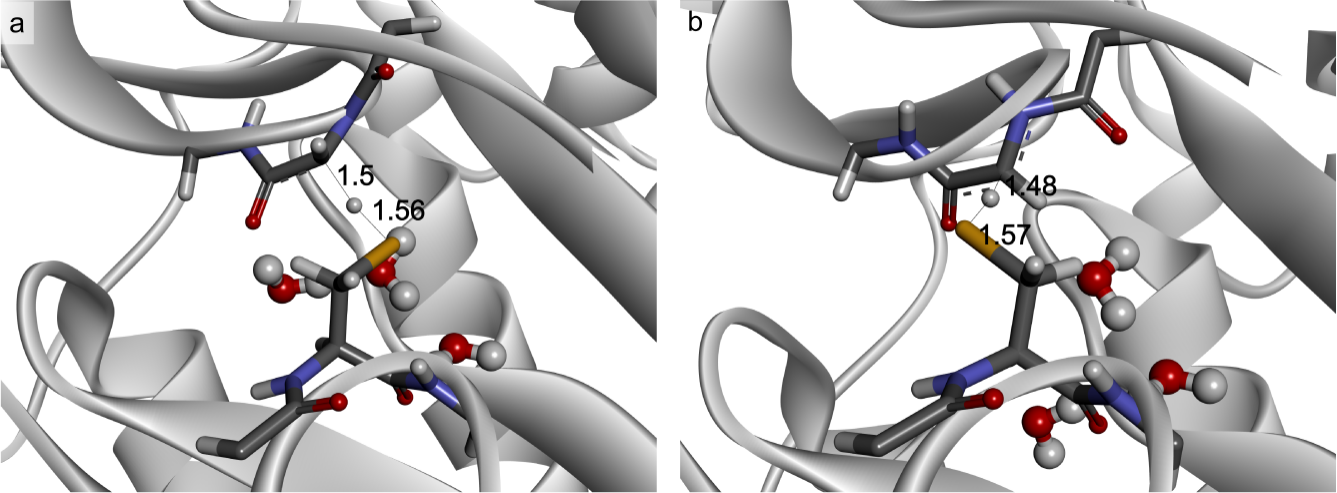
Geometry of the TS^apo^ for a) pro*R* and b) pro*S* conformation. Three water molecules in the vicinity of the active site are presented as balls and sticks models.

Due to the higher initial distance, the loop conformation has to change more significantly in order to enable efficient hydrogen transfer. After that, the geometry of the pro*R*-TS^apo^ shows distances of d(C–H) of 1.5 Å, d(S–H) of 1.56 Å, and an S–H–C angle of 159° (Figures 5a and S10), very similar to the values of the corresponding pro*R*-TS^ES^. The calculated spin densities are 0.49 at the Cα of Gly829 and 0.41 at the S atom of Cys493.

The pro*S* transition requires Cys493 to rotate to the pro*S* side of the G-loop which also has to bend in order to present its *si* face. This requires a stronger departure from the planarity of the Gly peptide bonds than in the case of pro*R* TS^apo^ (with a dihedral angle (N–Cα–C–N) of −40° with respect to −26° in pro*R* TS^apo^). Thus, the geometry of the pro*S* TS^apo^ is slightly more stretched than observed for the pro*R* transfer (Figure 5b, d(C–H = 1.49 Å, d(S–H)=1.57 Å, and S–H–C angle 171°) while the spin density is 0.46 at Cα of the Gly and 0.34 at S atom of the Cys. Furthermore, to reach the *si* face of the glycyl radical, Cys493 has to attain a higher energy conformation for pushing away the hydrophobic residues of Leu391 and Val709.

The energetics of both processes, when compared to the respective starting points (E^apo^), suggest that the pro*R*-TS**^apo^** is associated with a higher energy barrier (99.8 kJ/mol) than the pro*S*-TS**^apo^** (80.6 kJ/mol) (Tables 2, S8 and S9). The latter value, however, omits an additional required energy input of 86.8 kJ/mol for the conformational shift from pro*R* E^apo^ to pro*S* E^apo^. Therefore, this result strongly suggests a high preference for the pro*R* over a pro*S* hydrogen transfer in apo BSS, if it is possible at all. Furthermore, the thiyl radical intermediate (I^apo^) exhibits a higher energy than E^apo^ by 7.2 kJ/mol in the case of the pro*R* transfer and by approximately 78 kJ/mol in the case of the pro*S* transfer (although such a high energy of pro*S* I^apo^ seems to be associated with increased size of QM part, see Table S8). It should be noted, however, that the QM:MM methodology, with mostly frozen coordinates outside of the active site, is not the best tool for precise estimation of energy differences between different conformations of the protein.

When compared to the barriers for the E:S or E:P models, it appears that the presence of the reagent significantly facilitates the activation of Cys493. The barriers for the holoenzyme with either bound substrate or product are at least 40 kJ/mol lower compared to those of the apoenzyme.

As the analysis of TS^apo^ revealed the proximity of H_2_O molecules to the glycyl radical, we decided to investigate whether the H atom transfer from Cys493 could proceed with the assistance of water. In such a mechanism, a water molecule would donate one of its H atoms to the radical Gly, forming a transient hydroxyl radical which would immediately react with the sulfhydryl group of Cys493, yielding the thiyl radical. If feasible, such a mechanism would provide a potential alternative way for H/D exchange in the apoenzyme that may overcome the inferred conformational restraints. Our calculations showed that this mechanism is theoretically possible as we were able to locate the TS associated with the H_2_O molecule donating a H atom to radical Gly, with C–H and H–O distances of 1.36 and 1.41 Å and a C–H–O angle of 163° (see TS^apo-H2O^ at Figure S11), while forming weak H-bond interaction with the sulfhydryl group of Cys493. However, the calculations showed that such a process is associated with a prohibitively high energy barrier (228.5 kJ/mol). As a result, we regarded this pathway as highly improbable and did not further investigate it. The extensive investigation of the geometry for a concerted transfer of two H atoms (from H_2_O to glycyl radical and from SH group to H_2_O without formation of the transient OH radical) did not allow localization of the transition state of the first order and also exhibited prohibitively high energy.

### H/D Exchange at the Glycyl Radical

If the solvent of active BSS is exchanged for D_2_O, the EPR signal associated with the glycyl radical changes drastically to an isotropic spectrum without apparent hyperfine splitting.^62^ This occurs as well in PFL,^39,44^ but not in ARNR, where the hyperfine splitting does not change even after 12 h of incubation in D_2_O-based buffers.^61^ The observed spectral change for BSS indicates a complete exchange of the remaining protium hydrogen at Cα of the glycyl radical within the time required for changing the buffer (ca. 1 h).^62^ Since the hydrogen substituents of Gly829 are not acidic enough to spontaneously exchange with water as evident from experiments with model compounds,^63^ the process needs to be coupled to hydrogen transfer reactions with residues containing exchangeable hydrogens, such as the GRE activation/deactivation processes or the hydrogen transfer cascades between Gly829 and Cys493 associated with the reaction mechanism. GRE activation/deactivation appears unlikely to cause H/D exchange at the glycyl radical, as biochemical and structural data on PFL and its activating enzyme^31,36,39^ suggest that the generation of the glycyl radical by the respective activating enzyme is stereospecific. Moreover, a reversible glycyl radical quenching process has only been ascribed to PFL among the GRE, although it is disputed whether this is catalyzed by a special deactivase^79^ or by reaction with small molecules.^80^ In contrast, one of the two conserved Cys residues of the PFL active site is already known to be involved in the H/D exchange of the glycyl radical in PFL.^44^ Therefore, the observed H/D-exchange of BSS occurs most likely during the H-transfer reactions between Gly829 and Cys493, which occur in equilibrium in both directions. Because the sulfhydryl hydrogen of Cys493 is exchangeable with the solvent, this would lead to H/D exchange at the glycyl radical if the H-transfer reaction between Cys and Gly occasionally occurred in the less preferred enantiomeric orientation. Therefore, the observed H/D exchange at Cα of the glycyl radical can be explained if the energetic barriers of pro*R*- and pro*S*- directed hydrogen transfer from Cys493 to the glycyl radical are not too far apart, making the processes kinetically comparable.

Deuteration of certain positions influences the heights of the energetic barriers of the respective transition states of H/D exchange pathways through primary and secondary kinetic isotope effects (KIE). This means that we expect a primary KIE when a deuterium is transferred to or from Gly and a significantly smaller secondary KIE when a deuterium is bound to the Cα atom of Gly during a protium transfer. Combinations of primary and secondary effects are expected when a deuteron is transferred to or from a Gly already containing one or two bound deuteron(s). While any primary KIE always results in a rate reduction of the reactions with isotope-labeled substrate, secondary KIE may either enhance (“inverse effect”) or attenuate (“normal effect”) the rates, especially if the labeled atom changes its hybridization between sp^2^ and sp^3^ as in this case.^81^

Therefore, we analyzed all possible scenarios separately, i.e., when the H of the Gly829 radical is exchanged to D, when the D is exchanged back to H, and when Gly829 stays either fully protonated, half or fully deuterated, all in both possible geometries via the *re* or the *si* face of the radical Gly (Tables 2, S6 and S8). Moreover, we did these calculations with three different forms of BSS: the enzyme in complex with the substrates (E:S), with the products (E:P), or as an apoenzyme with an empty active site cavity (apo).

### Model for the E:S Complex

Fully protonated BSS showed barriers of 40.4 and 72.2 kJ/mol for the forward and reverse reactions of H exchange with Cys493 via the *re* face of the glycyl radical, but of 62.2 and 95.8 kJ/mol via the *si* face. Therefore, this hydrogen transfer appears to be highly enantiospecific for the *re* face, which proceeds over 5600 times faster than via the *si* face and in the reverse process over 12 000 faster for the pro*R*-H transfer compared to pro*S*.

**Figure 6:**
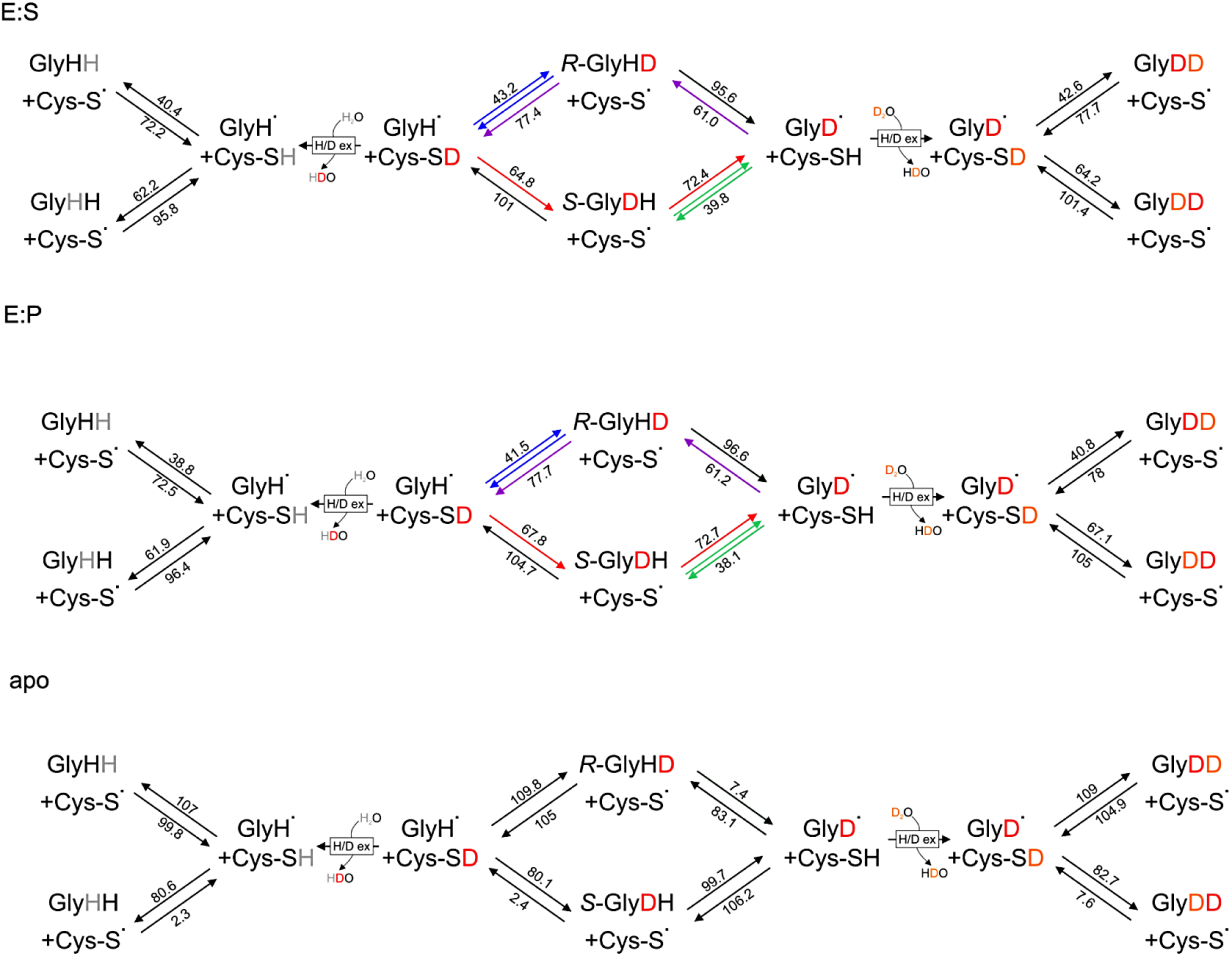
Schematic representation of H/D transfers for the E:S complex. The values indicate the energetic barriers of the respective transition states [kJ/mol]. The positions of the H and D atoms at Cα of Gly829 are assigned with the *si/S* position left and the *re/R* one right).

The transfer of deuterium from Cys493 to the *re* face of the glycyl radical is associated with a barrier of 43.2 kJ/mol (Figure 6 E:S), representing a slight increase by 2.8 kJ/mol compared to that for H/H transfer, which results in an intrinsic kinetic isotope effect (KIE) of 3.0. The reverse reaction, i.e., the transfer of a D atom from *R*-GlyHD back to the thiyl radical, proceeds with a barrier of 77.4 kJ/mol. The predicted intrinsic KIE associated with this transfer is higher than in the forward process (7.9), due to a higher increase of the energy barrier (5.2 kJ/mol) upon replacing protium for deuteron.

If deuterium is transferred from Cys493 to the *si* face of the glycyl radical, the barrier is at 64.8 kJ/mol, representing an increase of 2.6 kJ/mol compared to that of the *si* H/H exchange. The reverse reaction, the backward transfer of a D atom from the S-enantiomer of monodeuterated Gly829, occurs with a barrier of 101.0 kJ/mol, 5.2 kJ/mol more than for the *si* H/H exchange (Figure 6).

The barriers for both hydrogen transfer reactions from Cys493 to the deuterated glycyl radical turned out to be slightly lower than those for the corresponding H/H exchange reactions due to an inverse secondary KIE: the barriers for protium transfer to the *re* and *si* faces are 39.8 and 61 kJ/mol (Figure 6), which are 0.6 and 1.2 kJ/mol lower than the respective values for the H/H transfer and the associated inverse KIE amount to 0.78 and 0.63, respectively. Similarly, the secondary KIE also facilitates deuteron transfer when the glycyl radical is already deuterated (GlyD^•^). Such a process is associated with a barrier of 42.6 kJ/mol for the *re* face attack and 61.0 kJ/mol for the *si* face attack. (Figure 5), resulting in a lower intrinsic KIE with respect to deuteron transfer to a nondeuterated glycyl radical (decrease from 3.0 for GlyH^•^ to 2.24–2.35 for GlyD^•^). Thus, it is easier to transfer a second deuteron if the first one is already present at the glycyl radical.

In the reverse process, the hydrogen transfer from *R*-GlyHD to the thiyl radical occurs with a barrier of 95.6 kJ/mol, which is 0.2 kJ/mol lower than for H/H (KIE 0.9), while the barrier for hydrogen transfer from *S*-GlyDH is at 72.4 kJ/mol, i.e., 0.2 kJ/mol higher than for the respective H/H exchange (KIE 1.1) (Figure 6). The removal of a deuteron from GlyD_2_ is significantly slower compared to the process when only protium is involved, and the energy difference of 5.5 kJ/mol results in a KIE of 9 (for values see Tables 2 and S6 and Figure 6).

The entire scheme of all possible H and D exchange reactions in the BSS is shown in Figure 6. The calculated barriers indicate immediately that the conservative exchange reactions resulting in the retention of either H or D in the glycyl radicals are highly favored over those leading to isotope exchange. Judging from the much lower barriers for both the forward and reverse reactions in the case of GlyH^•^, this process (with deuterated Cys493) occurs mostly via the *re* face (labeled by blue arrows in Figure 6), whereas in the case of GlyD^•^, it occurs mostly via the *si* face (with protiated Cys493, labeled green in Figure 6). To obtain any exchange of H to D (or the reverse) in the glycyl radical, some of the less favorable reactions in the other stereochemical orientation have to occur. For exchanging H to D, the more favorable pathway is the *si*-face-directed attack of deuterated Cys493 combined with the transfer of the pro*R*-protium from the *S*-glycyl intermediate back to the thiyl radical (red arrows in Figure 6). Although the barrier of the first step is significantly higher than that for the *re* attack (64.8 vs 43.2 kJ/mol, indicating a 5400-fold slower rate), the second step of protium vs deuterium transfer is highly favored (barriers 72.4 vs 101 kJ/mol), while the alternative pathway via *R*-Gly829 contains a prohibitively high barrier at the second step (95.6 kJ/mol).

In conclusion, the very high barrier of removing H from *R*-GlyHD (95.6 kJ/mol) suggests that deuterium exchange is correlated with the rare *si*-directed hydrogen transfer events, which produce an *S*-GlyDH intermediate (Figure 6 red pathway). The calculated back-transfer of H from *S*-GlyDH is much faster than that of D (difference of the barriers = 28.6 kJ/mol), so an H/D exchange at the glycyl radical will be inevitable if the first step occurs.

### Prediction of Deuteration Kinetics for E:S

The prediction of the elementary kinetic constants (Table 3) allows the estimation of the expected rates of different pathways of the H/D exchange process.

**Table 3:** Prediction of Elementary Kinetic Constants (at 303 K) and Values of the Intrinsic KIE (iKIE) for the E:S Complex Model

system	transfer	Δ(E+thermal) [kJ/mol]	k [s^–1^]	iKIE
E:S *re* attack	*re* H GlyH^•^	40.4	6.8 × 10^5^	1.0
	*re* D GlyH^•^	43.2	2.3 × 10^5^	3.01
	*re* H GlyD^•^	39.8	8.7 × 10^5^	0.78
	*re* D GlyD^•^	42.6	2.9 × 10^5^	2.35
E:S *si* attack	*si* H GlyH^•^	62.2	122	1.0
	*si* D GlyH^•^	64.8	42	2.89
	*si* H GlyD^•^	61.0	192	0.63
	*si* D GlyD^•^	64.2	54	2.24
E:S reverse *R*-transfer	*R*-H GlyH_2_	72.2	2.3	1.0
	*R*-D *R*-GlyHD	77.4	0.29	7.87
	*R*-H *S*-GlyDH	72.4	2.1	1.12
	*R*-D *R*-GlyD_2_	77.7	0.25	9.09
E:S reverse *S*-transfer	*S*-H GlyH_2_	95.8	1.9 × 10^–4^	1.0
	*S-*D *S*-GlyDH	101.0	2.4 × 10^–5^	7.75
	*S*-H *R*-GlyHD	95.6	2.1 × 10^–4^	0.91
	*S*-D *R*-GlyD_2_	101.4	2.1 × 10^–5^	8.9

First, we compared the calculated rates for the preferential pathway via *S*-Gly829 (red arrow in Figure 5) with those of the alternative pathway via *R*-Gly829. It turns out that the value of k^S^_H/D_ is calculated as 2 s^–1^ while that of k^R^_H/D_ equals 2.1 × 10^–4^ s^–1^, which means that the pathway proceeds exclusively through *S*-GlyDH while *R*-GlyHD is of no consequence. This shows that despite the 5400-fold kinetic preference for the transfer of D via the *re* face, one of the expected rare D transfers via the *si* face of the glycyl radical is a prerequisite for the H/D exchange.

The same approach can be applied to analyzing the experiment when the deuterated enzyme is incubated in H_2_O. For the backward exchange of D to H in the glycyl radical, the preferred pathway proceeds via the *re* face attack (purple arrows in Figure 6) with a calculated value of k^R^_D/H_ of 0.29 s^–1^, while the alternative pathway, starting with the transfer of H to the *si* face, is associated with a k^S^_D/H_ value of 2.4 × 10^–5^ s^–1^, almost 12 000-fold slower than the process via the *re* face.

Comparing the relative rates of preferred pathways for exchanging either H to D or D to H in the glycyl radicals (k^S^_H/D_ of 2 s^–1^ vs k^R^_D/H_ of 0.29 s^–1^), we obtain a ratio of 6.7, suggesting a slightly faster rate of deuterating the glycyl radical in D_2_O, compared with reprotonation in H_2_O. However, the relative ratio indicates that both processes should be observable within approximately the same experimental time frame.

### Model for the E:P Complex

The calculations for the E:P complex indicate differences of only 4 kJ/mol or less from the respective energy barriers of the E:S complex (Tables 2 and S6). Therefore, the pattern of favorable and unfavorable H or D transfer reactions is identical to that of the E:S complex with only minor differences in the ratios of the respective rates. In particular, the difference of the energy barriers for the transfer of D from Cys-SD to GlyH^•^ between the pathways via the *re* and *si* face is 26.4 kJ/mol which translates to a 34 200 faster rate of the former process and a very high enantioselectivity (Table S10). As in the case of the E:S complex, the kinetic analysis reveals that the exchange of protium to deuterium in the glycyl radical via *S-*Gly829 (k^S^_H/D_) is almost 11 500 times higher (1.6 s^–1^) than via *R-*Gly829 (k^R^_H/D_ = 1.4 × 10^–4^ s^–1^), while in the backward exchange of deuterium to protium, the process via *R*-GlyHD is preferred (k^R^_D/H_ = 0.26 s^–1^) compared to that via *S*-GlyDH (k^S^_D/H_ = 5.7 × 10^–6^ s^–1^). Similarly, as in the case of the E:S complex, the estimated rate of enzyme deuteration in D_2_O turns out to be 6.2 times higher than that for backward protonation of the deuterated enzyme in H_2_O.

### Model for the Apoenzyme

In the apoenzyme, the situation is seemingly reversed than in the case of the E:S and E:P complexes. Our calculations indicate a preference for H transfer to the *si* over the *re* face of the glycyl radical (barriers of 80.6 kJ/mol vs 107 kJ/mol), which would indicate an almost 40 000 faster rate of *S*-GlyDH formation (Table S11). However, we must remember that those barriers are calculated with respect to the E^apo^ conformational states, which were derived from an internal reaction coordinate scan started at the *si-* or *re*-oriented TS^apo^. As a result, the conformational changes enforced during the optimization of the TS may persist in the E^apo^ or I^apo^ states. It turned out that the energy of the pro*S* conformer of E^apo^ is 86.8 kJ/mol higher than that of the pro*R* conformer. Such a situation was not observed in the case of the E:S and E:P models, where the energy differences between the respective reference states were minimal.

Therefore, it should be concluded that, based on our calculations, any H/D exchange between Cys493 and Gly829 of the apoenzyme would be highly enantioselective and would not easily exchange the H atom at Gly along the depicted mechanism. The predicted kinetic constants (7.3 × 10^–7^ vs 1.6 × 10^–18^ s^–1^) show that the favored H/D exchange pathway in apo-BSS proceeds through *R*-GlyHD, regardless whether the barriers of the pro*S* process need to be increased by 86.8 kJ/mol because the higher energy level of this conformant. However, even the favored H/D exchange pathway in apo-BSS would be 2.67 × 10^6^ -fold slower than that in the E:S or E:P complex. This equals to one predicted exchange per 378 h, practically precluding H/D exchange in the absence of bound substrate or product.

### Reinterpretation of the EPR Studies on BSS

**Figure 7:**
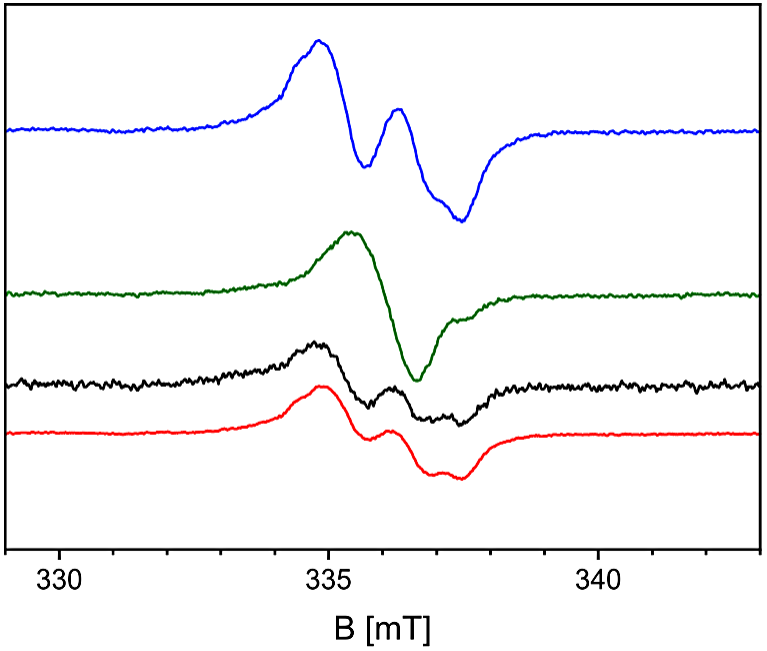
Electron paramagnetic resonance (EPR) analysis of the H/D exchange reactions at the glycyl radical of BSS. An anaerobically prepared cell-free extract of toluene-grown cells of *Aromatoleum toluolicum* was used, either as prepared (blue), after exchanging the solvent to D_2_O (green), and after exchanging it back to H_2_O (black). The red curve approximates the black spectrum by adding the blue and green spectra at a ratio of 0.44:0.21, which fits well to a 40% loss of radical content in this sample, as calculated by spin quantitation. Note that the amplitude-based 68:32 ratio shown here is converted to an 80:20 ratio when analyzed based on the spectrum integrals.

After revisiting the EPR experiments performed on the H/D exchange reactivity of BSS,^62^ we noticed that the signal changed almost completely from a protium- to a deuterium-containing glycyl radical upon changing the solvent from H_2_O to D_2_O but appeared to revert only partially when the buffer was changed back to H_2_O, resulting in a mixed signal (Figure 7).

We quantitated the contributions of the spectra of the protium- and deuterium-containing enzymes on the basis of the integrated original spectra, indicating that 80% of the active BSS molecules had reverted to the protium state, while 20% still contained the deuterated glycyl radical (Figure 7 red curve). Since the buffer exchange procedures lasted approximately 1 h in either direction, this observation correlates well with the postulated faster rate of exchanging protium to a deuteron at the glycyl radical than that of the reverse process. In this respect, it is noteworthy that the EPR experiments on BSS or PFL were always performed in cell extracts that contained substrates or products, while EPR on ARNR was performed in the presence of formate but in the absence of nucleoside triphosphates.^57,62,82^

The calculated energy barriers for H/D exchange in E:S, E:P and apo BSS indicate that the exchange from protium to deuterium in the glycyl radical occurs at an approximately 7-fold higher rate in E:S and E:P than the backward exchange of the deuterium to the protium. In the apoenzyme, we calculated the inverse situation, with a 4.3-fold slower rate of protium to deuterium exchange compared to the reversed process. However, if the rates of E:S and E:P and apo are compared, we find very similar rates for E:S and E:P complexes, but the calculated rates for the apoenzyme are more than 5 orders of magnitude slower. These findings are in qualitative agreement with the results of the isotope exchange experiments monitored by EPR spectroscopy. If we assume that the time needed for preparation only allowed for partial exchange of deuterated glycyl radical with protium, we would expect full exchange of protium by deuterium because of the higher rate in the case of E:S or E:P complexes. In the case of apo BSS, the calculated rate was so low that any H/D exchange would not be observable in a reasonable time.

### Experimental Support for H/D Exchange at Cys493

**Figure 8:**
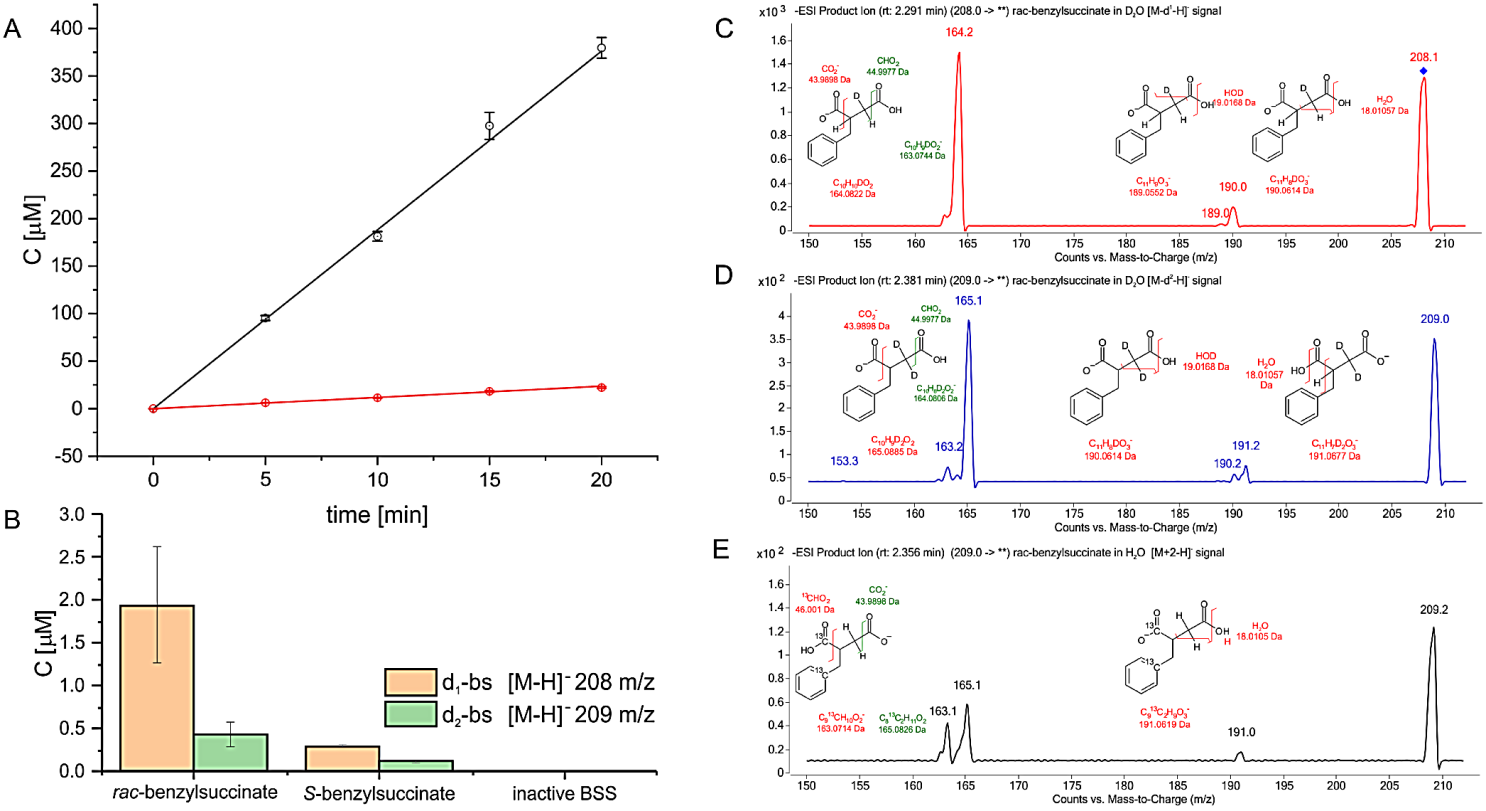
Results of the H/D exchange experiments. A) D/H exchange during reaction with *d*_8_-toluene in H_2_O, black line–formation of *d*_8_-benzylsuccinate (215 *m*/*z*), red line formation of *d*_7_-benzylsuccinate (214 *m*/*z*); B) yields of labeled products after 4h of benzylsuccinate incubation with BSS in D_2_O, C) product ion spectrum of 208 *m*/*z* (mixture of *d*_1_-benzylsuccinate and ^13^C-benzylsuccinate) D) product ion spectrum of 209 *m*/*z* (mixture of *d*_2_-benzylsuccinate, d_1_-^13^C_2_-benzylsuccinate and ^13^C_2_-benzylsuccinate), E) product ion spectrum of 209 *m*/*z* from the assay with inactive BSS (fragment ions only from^13^C_2_-benzylsuccinate). Error bars–standard deviation.

To gain insight into the rate of H/D exchange at the Cyr493 SH group, we have conducted two types of isotope-labeled experiments. First, we followed what products were formed in the reaction of fumarate with *d*_8_-toluene in H_2_O, detecting *d*_8_-benzylsuccinate and *d*_7_-benzylsuccinate by MS. If no H/D exchange occurred at the SH group, we would expect only the *d*_8_-product to be formed. The rate of formation of the *d*_7_-product (with one deuteron from toluene replaced by a proton from the reaction environment) revealed the rate of SD/SH exchange during the reaction. We indeed detected the simultaneous appearance of both products, i.e., d_8_- and *d*_7_-labeled benzylsuccinate. The specific activity of *d*_8_-benzylsuccinate formation turned out to be 2.4 nmol min^–1^ [mg extract]^−1^ while that of *d*_7_-benzylsuccinate formation was 0.15 nmol min^–1^ [mg extract]^−1^, indicating a 16-fold lower rate of H/D exchange compared to the overall reaction rate. Figure 8

The second experiment was conducted in 40% D_2_O and the presence of the product benzylsuccinate. We were monitoring isotope enrichment by enzyme-catalyzed H/D-exchange into the product, following the quasimolecular [M-H]^−^ ions of benzylsuccinate (207 *m*/*z*), *d*_1_-benzylsuccinate (208 *m*/*z*) and *d*_2_-benzylsuccinate (209 *m*/*z*) (Figure 7B). As control experiments, we used an oxygen-inactivated cell extract and a sample enriched with (*S*)-benzylsuccinate. After correcting the recorded signals for isotope intensities from^13^C (i.e., [M+1-H]^−^ and [M+2-H]^−^), we were indeed able to detect small peaks confirming H/D exchange into benzylsuccinate. The signals were unequivocally detectable after 4 h of incubation when we recorded the presence of 1.94 ± 0.28 μM of d_1_- and 0.44 ± 0.06 μM of *d*_2_-benzylsuccinate representing specific activities of 1.0 and 0.3 pmol min^–1^ [mg extract]^−1^, respectively. This indicates that the rate of D/H exchange at the SH group of Cys493 is approximately 100 times faster during the forward reaction of benzylsuccinate formation than during prolonged incubation with the product. Notably, we have detected only 0.3 μM d_1_- and 0.1 μM of d_2_-benzylsucciante in the control sample with predominantly (*S*)-benzylsuccinate, which most probably comes from the exchange in (*R*)-benzylsuccinate still present in the cell extract, although we cannot exclude that the enzyme can catalyze such exchange for the *S* enantiomer. The double deuteration of the product can be explained by the enantio-unspecific exchange of the second proton at the C3 methylene group of benzylsuccinate. After the first deuteration, the enantiospecificity of the process should decrease due to the internal kinetic isotope effect.

Furthermore, the analysis of the MS fragmentation pattern confirmed C3 of benzylsuccinate as the position of both transferred deuterons. The standard fragmentation of the benzylsuccinate ion (207 *m*/*z*) consists of the removal of an OH^–^ group from the carboxylic group and a proton from the adjacent C3-carbon atom, yielding water and a fragmentation ion of [*M*-18-H]^−^ at 189 *m*/*z* (Figure S12). If the C3 atom is substituted by one or two deuterium atoms, we can expect an additional peak to appear at a mass of [*M*-19-H]^−^, indicating the removal of DOH. The product ion spectrum of the monodeuterated benzylsuccinate peak at 208 *m*/*z* (Figure 7C) already exhibits traces of such an ion at 189 *m*/*z* along with the more intense one of 190 *m*/*z* corresponding to [*M*-18-H]^−^ fragmentation. This is even more apparent in the analysis of the mass peak of 209 *m*/*z*, where the [*M*-19-H]^−^ peak of 190 *m*/*z* amounts to half of the size of the one of 191 *m*/*z* (Figure 7D). The corresponding changes in the patterns of the fragmented ions generated by decarboxylation additionally confirm the identification of the respective mono- and bideuterated products (see Supporting Information for more detailed description).

## Discussion

In this study, we present a theoretical modeling study of the mechanistic pathway describing how the intermediary thiyl radical is formed in BSS by reversible H transfer between the active site Cys and the glycyl radical. This represents the first time this reaction has been assessed in the full geometric context of any GRE. Previous computational studies on the mechanism of BSS either did not include this step^78^ or used a gas-phase model containing only small fragments of the Cys and Gly residues and modeled every transition reaction separately.^83^ Because of the methodical limitations, the gas-phase model produced essentially the same parameters for each reaction type in a symmetrical scheme, which does not allow one to describe the influences of the enzyme, like conformational changes of the active site, disparity of forward and backward reactions, enantioselectivity of the process, or the effects of bound substrate or product. The reported TS barrier of this previous gas-phase study for H transfer between Cys and the glycyl radical was at 44.8 kJ/mol, and the thiyl radical intermediate showed a higher energy than the starting point by 14 kJ/mol.^83^

Our calculations indicate that the gas phase model is inadequate to reflect the actual reaction mechanism in the following points: (i) the influence of bound substrates or products, (ii) the actual energy differences between transition states and intermediates, (iii) different TS energy levels for forward and backward reactions, (iv) strong enantiospecificities, and (v) isotope effects of the transfer reactions.

(i) Conversion of Cys493 of BSS to a thiyl radical proceeds significantly faster when the conformation of the enzyme is adjusted to binding the substrates or the product compared to apo-BSS with an empty active site. The energy difference of the respective TS of 40 kJ/mol (i.e., the difference between pro*R* TS^E:S^ and pro*S* TS^apo^) translates into an 8.6 × 10^6^ times faster H-transfer rate in the substrate- or product-bound state of the enzyme than in the apoenzyme, effectively precluding this reaction in apo-BSS.

(ii) In contrast to the gas phase model, the thiyl radical intermediate turns out to be more stable than the substrate or product bound starting state in the respective models. A similar situation of the thiyl intermediate shows slightly higher energy than the glycyl radical state (respectively significantly higher for the strained pro*S* conformation) was only obtained for apo-BSS. The calculated values suggest that the equilibrium of H transfer is shifted toward maintaining the glycyl radical state in the apoenzyme, while Cys493 should be predominantly in the thiyl radical state in the substrate- or product-bound states, thus facilitating the catalytic reaction. We may assume that the enzyme changes its confirmation upon product release, so the relative energy changes, and the equilibrium shifts back to that of the glycyl radical state. This may help to safeguard the enzyme from accidental proteolysis, oxygen-dependent cleavage, or other side reactions.

(iii) Forward and backward reactions of the same transition are not equivalent in any of the full enzyme models, due to stabilization of the radical cysteine state in holoenzyme. Rather, we observe usually very different TS energies for these processes, which may help in guiding the reaction pathway of the overall reaction of BSS.

(iv) All states, apo-BSS, the E:S and E:P complexes, exhibit strong enantioselectivity for the hydrogen transfer process between Gly829 and Cys493. In the substrate- or product-bound states, the *re* side of radical Gly829 is more prone to attack by Cys493, but the attack from the *si* side is still kinetically feasible.

(v) Isotope effects are predicted to be significant for exchanging the protium atoms of the glycyl radical, resulting in an easier H/D exchange process at Gly of the protium by deuterium than vice versa. Combining these calculations with those on the enantioselectivity of the reaction, we predicted that BSS should have a low, but detectable, isotope exchange activity with the product benzylsuccinate and provided an explanation for the observed H/D exchange of the glycyl radical.

We show experimental support for the predicted H/D transfer reactions in the product-bound state of BSS, since the enzyme catalyzes H/D exchange at the carboxymethyl side chain of the product benzylsuccinate (C3 atom). As expected, this reaction depends on the active, radical-containing state of BSS and even proceeds sequentially to exchange both protium atoms of C3 of benzylsuccinate. In addition, we identified a D/H exchange activity during the BSS reaction with *d*_8_-toluene by confirming small amounts of *d*_7_-benzylsuccinate as a byproduct of abundant *d*_8_-benzylsuccinate, which confirms H/D exchange at Cys493.

The kinetically favored H-transfer pathways between Cys493 and Gly829 in either model result in the retention of the same H atom in the glycyl radical, contradicting experimental evidence. Therefore, an occasional kinetically unfavorable transfer step to the *si* side needs to occur, which leads to the observed H/D isotope exchange reactions of the glycyl radical. The same principles were applied for the H/D-transfer reaction in substrate- and product-bound enzymes, while in contrast, the calculated rates for apo-BSS suggest that neither H transfer to nor H/D exchange of the glycyl radical should be observable in a reasonable time frame because of their slow kinetics.

This predicted behavior indeed fits the respective experimental conditions since all EPR experiments with BSS or other FAE to date were conducted in cell extracts,^57,62,82^ which always contain enough product and leftover substrates to saturate the enzymes (e.g., from the growth of the cells on toluene). This suggests that the observed change of the glycyl radical signal in EPR spectra upon incubation in D_2_O is facilitated by the binding of either of the substrates of the product. This would allow BSS to attain a conformation that exhibits lower H-transfer barriers from Cys493 to the glycyl radical and speeds up the H/D exchange process.

Our calculations also indicated that the exchange of H to D in the glycyl radical of BSS (in D_2_O) occurs significantly faster than the reverse exchange of D back to H (after changing D_2_O to H_2_O). This prediction is consistent with the observed complete exchange of H to D in the glycyl radical within the time needed for sample preparation, while the assumed 6.4-fold slower reverse exchange of D to H was apparently still ongoing after the same preparation time. However, this observation may also indicate a partial loss of the enzyme-bound substrates/products during the buffer changing procedure, which only affects the observed results after two consecutive buffer exchange steps.

Looking at other GRE for which H/D exchange experiments have been reported, the samples of purified and in vitro-activated PFL contained 2 mM substrate or substrate analog (pyruvate or oxamate) as a necessary component of the activation reaction.^39^ Moreover, the covalently bound pyruvate in PFL is attached at Cys418, enabling the presence of bound substrate during the Cys419-mediated H/D exchange at the glycyl radical Cα atom.^44^ In contrast, ARNR was essentially assayed in the apoenzyme state, i.e., with only formate added as a cosubstrate for the activation reaction, but without nucleotide triphosphate, which was correlated with the absence of observable H/D exchange.^61^

## Conclusions

In this study, we show by computer modeling that activated BSS containing a radical on Gly829 (1) requires bound substrates or product to enable H transfer or H/D exchange between Cys493 and the glycyl radical while these reactions are precluded in apo-BSS; (2) retains the H- or D atoms of the glycyl radicals when acting with the preferred stereospecificity, but (3) is able to initiate H/D exchange into the glycyl radical at a slow rate (as well as even slower reverse D/H exchange) by occasional reactivity in the nonpreferential stereospecificity, together with (4) some experimental support for these predictions. The behavior of the enzyme reported here may be valid for all GRE, indicating that substrate or product binding may be a prerequisite for both H transfer between the active site Gly and Cys residues and the reactions involved in the H/D exchange of the glycyl radical. Since BSS has so far been only investigated in the naturally activated state in cell extracts while in vitro activation is difficult and has only recently been accomplished,^30^ a broader data basis with biochemically better accessible GRE will be very helpful to further investigate the alleged requirements proposed in this study.

## Supporting Information Available

Additional information on methods (EPR, LC-MS/MS, MD and QM:MM), analysis of the fumarate binding pocket, RMSD for MD simulations, additional results on MD analyses and QM:MM modeling, figures of all stationary states, additional chromatograms, and detailed description of product fragmentations (PDF)
PDB geometries of all structures discussed in the paper (ZIP)
AMBER parameters for radical residues and reagents used in the simulation (ZIP)
