# Supplementary material for: Modeling the Initiation Phase of the Catalytic Cycle in the Glycyl-Radical Enzyme Benzylsuccinate Synthase
Source: J Phys Chem B. 2024 Jun 7;128(24):5823–39. doi: 10.1021/acs.jpcb.4c01237 (PMC11194802; doi:10.1021/acs.jpcb.4c01237)
Supplement: Supplementary file 1 — jp4c01237_si_001.pdf [file jp4c01237_si_001.pdf]

Supporting Information

Modeling the Initiation Phase of the Catalytic Cycle in the Glycyl-Radical Enzyme Benzylsuccinate Synthase

Maciej Szaleniec[1]*, Gabriela Oleksy[1,3], Anna Sekuła[1], Ivana Aleksić[1], Rafał Pietras[2], Marcin Sarewicz[2], Kai Krämer[3], Antonio J. Pierik[4], Johann Heider[3,5]*

1 – Jerzy Haber Institute of Catalysis and Surface Chemistry, Polish Academy of Sciences, 31-201, Kraków, Poland; email: maciej.szaleniec@ikifp.edu.pl

2 - Department of Molecular Biophysics, Faculty of Biochemistry, Biophysics and Biotechnology, Jagiellonian University, Kraków, Poland

3 - Department of Biology, Laboratory for Microbial Biochemistry, Philipps University Marburg, 35043 Marburg, Germany;

4 - Biochemistry, Faculty of Chemistry, RPTU Kaiserslautern-Landau, D-67663 Kaiserslautern, Germany

5- Synmikro-Center for Synthetic Microbiology, Philipps University Marburg, 35043 Marburg, Germany; email: heider@staff.uni-marburg.de

Index

Methods S2

- EPR measurements S2
- LC-MS/MS methods S2
- Calibration curves for LC-MS/MS and LC-DAD benzylsuccinate quantitation S3
- MM geometry minimization protocol after MD S4
- Atom mask used in clustering S4
- QM of the model with H_2O S5

Results S5

- Fumarate binding pocket S5
- MD simulations S6
- QMMM calculations S10
- Prediction of elementary rate constants S19
- The geometries of the stationary points S21
- UHPLC-MS/MS product ion spectra S28
- Product ion fragmentation analysis S29

Methods

EPR measurements

The EPR spectrum confirming H/D exchange in D_2O at glycyl radical in BSS isolated from *Aromatoleum* sp. was recorded according to the following procedure. The X-band CW-EPR spectra were measured at 200 K using Bruker Elexsys E580 spectrometer and SHQ4122 resonator equipped with ESR900 cryostat (Oxford Instruments). Parameters were as follows: microwave frequency: 9.388 GHz; microwave power: 0.6 mW; modulation amplitude and frequency: 0.4 mT and 100 kHz, respectively; time constant: 20 ms; sweep time: 42 s; sweep width: 30 mT; center field: 334.6 mT.

LC-MS/MS methods

Table S1 Parameters of jet-stream ESI ion source used in the LC-MS/MS analysis.

Parameter	Value (+)	Value (-)
**Gas Temp [°C]**	300	300
**Gas Flow [L/min]**	10	10
**Nebulizer [psi]**	45	45
**Sheath Gas Heater [°C]**	300	300
**Sheath Gas Flow [L/min]**	10	10
**Capillary [V]**	3500	3500
**V Charging [V]**	500	1000

Table S2. Parameters of MRM method for the analysis of d^7-benzylsuccinate and d^8-benzylsuccinate.

Compound	Precursor ion [m/z]	Product ion [m/z]	Dwell [ms]	Fragmentor [V]	Collision energy [v]	Cell accelerator [V]	Polarity
**d_8-benzylsuccinate**	215.0	171.0	200	107	10	4	negative
**d_7-benzylsuccinate**	214.0	170.0	200	107	10	4	negative

Table S3. Parameters of Single Ion Monitoring (SIM) method for the analysis of D/H exchange in benzylsuccinate in D_2O.

Compound	Signal m/z	Dwell [ms]	Fragmentor [V]	Collision energy [v]	Cell accelerator [V]	Polarity
**benzylsuccinate**	207.0	200	107	10	4	negative
**M+1 benzylsuccinate**	208.0	200	107	10	4	negative
**M+2 benzylsuccinate**	209.0	200	107	10	4	negative

Table S4. Parameters for analysis of product ions of benzylsuccinate 207. 209, 209 m/z signals (respectively, $[M-H]^-$, $[M+1-H]^-$ and $[M+2-H]^-$).

**Precursor ion [m/z]**	**MS2 from**	**MS2 to**	**Scan time [ms]**	**Fragmentor [V]**	**Collision energy [v]**	**Cell accelerator [V]**	**Polarity**
207.0	150.0	212.0	500	107	10	4	negative
208.0	150.0	212.0	500	107	10	4	negative
209.0	150.0	212.0	500	107	10	4	negative

Calibration curves for LC-MS/MS and LC-DAD benzylsuccinate quantitation

A)

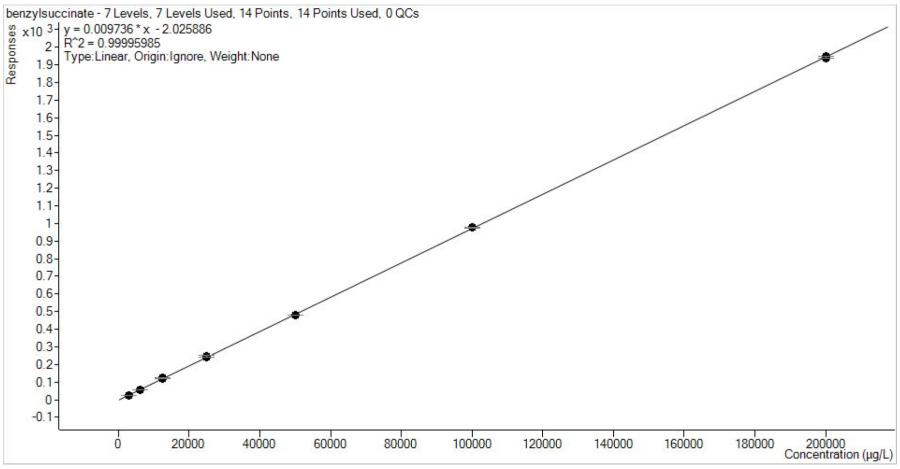

B)

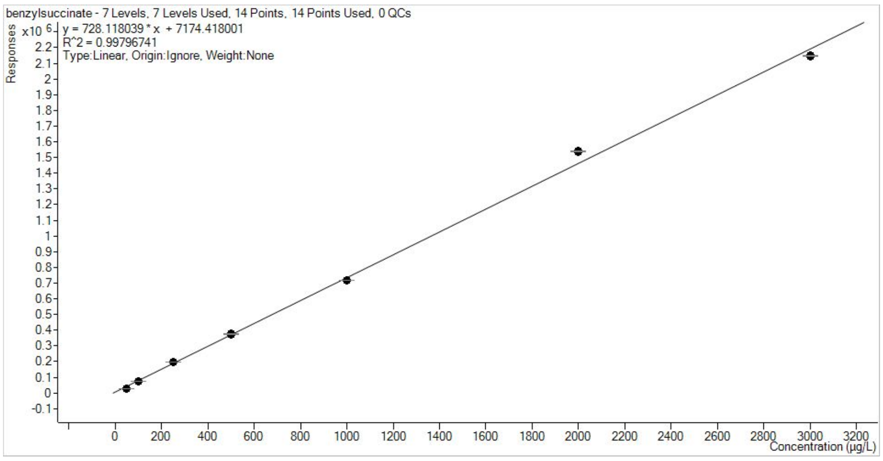

Figure S1. Calibration curves for LC-MS/MS quantitation of A) LC-DAD quantitation of benzylsuccinate; B) benzylsuccinate in SIM mode. Concentrations are given in µg/L.

MM geometry minimization protocol after MD

1. Minimization of geometry of water solvent (imin=1, maxcyc=5000, ncyc=500, ntb=1, ntr=1) with protein residue frozen with force 500
2. Minimization of geometry (imin=1, maxcyc=5000, ncyc=500, ntb=1, ntr=1) with protein residue frozen with force 10
3. Minimization of geometry (imin=1, maxcyc=10000, ncyc=500, ntb=1,) without any constrains

Atom mask used in clustering

E:S complex

:184@CA,CB,CG,CD:192@CA,CB,CG,CZ,OH:193@CA,CB,CG,ND2:194@CA,CB,OG:323@CA,CB,OG:376@CA,CB,CG,CZ,OH:379@CA,CB,CG1,CG2,CD1:380@CA,CB,CG,CZ:386@CA,CB,CG:485@CA,CB,CG,ND2:486@CA,CB:487@CA,CB,CG:488@CA,CB,SG:489@CA,CB,CG,SD:490@CA,CB,OG:491@CA,CB,CG,CD,N:500@CA,CB,CG,ND2:503@CA,CB,CG,CD,NE:506@CA:507@CA:508@CA:509@CA,CB,OG:511@CA,CB,CG,CZ:608@CA,CB,CG,CD1,NE1,CZ2,CH2,CZ3,CE3,CD2:609@CA,CB,CG,ND1,ND2,CE1:610@CA,CB,CG,ND2:612@CA,CB,CG1,CG2,CD1:700@CA,CB,OG1,CG2:702@CA,CB,CG,CD,NE2:703@CA,CB:704@CA,CB:705@CA:822@CA,CB:823@CA,CB,OG:824@CA:825@CA,CB,CG,CZ,OH

Apoenzyme

:177@CG1,:180@C,:181@N,CA,CB,CG,CD,OE1,OE2,CO,:182@N,CA,CB,C,O,:184@N,CA,CB,OG,:187@OG,:188@C,:189@N,CA,CB,CG,CD1,CE1,CZ,OH,CE2,CD2,C,O,:190@N,CA,C,O,:191@N,CA,CB,OG,:318@CZ,OH,CE2,:319@C,O,:320@N,CA,CB,OG,C,O,:321@N,CA,C,:369@CB,CG,CD,CE,NZ,:373@N,CA,CB,CG,CD1,CE1,CZ,OH,CE2,CD2,C,O,:376@N,CA,CB,CG2,CG1,CD1,C,O,:377@N,CA,CB,CG,CD1,CE1,CZ,CE2,CD2,:382@CG,OD1,OD2,C,O,:383@N,CA,CB,CG,CD1,CD2,C,O,:384@N,CA,C,:385@N,CA,CB,CG2,CG1,CD1,C,O,:482@CA,CB,CG,ND2,C,O,:483@N,CA,CB,CG1,CG2,C,O,:484@N,CA,CB,CG,CD1,CD2,C,O,:485@N,CA,CB,SG,C,O,:486@N,CA,CB,CG,SD,CE,C,O,:487@N,CA,CB,OG,C,O,:500@CB,CG,CD,NE,CZ,NH1,NH2,:503@C,O,:504@N,CA,C,O,:505@N,CA,C,O,:506@N,CA,CB,OG,C,O,:507@N,CA,C,O,:508@CG,CD1,CE1,CZ,CE2,CD2,:605@CB,CG,CD1,NE1,CE2,CZ2,CH2,CZ3,CE3,CD2,C,O,:606@N,CA,ND1,CE1,NE2,C,O,:607@N,CA,CB,CG,OD1,ND2,C,O,:608@N,CD,CA,C,O,:609@N,CA,CB,CG2,CG1,CD1,C,O,N,:697@N,CA,CB,CG2,OG1,C,O,:698@N,CA,C,O,:699@N,CA,CB,CG,CD,OE1,NE2,C,O,:700@N,CA,CB,C,O,:701@N,CA,CB,CG1,CG2,C,O,:702@N,CA,C,O,:703@N,CA,CG,CD2,:706@CD,OE2,:758@CB,CG,CD1,:820@CB,OG,:821@N,CA,C,O,:822@N,CD1,CE1

QM of the model with H_2O

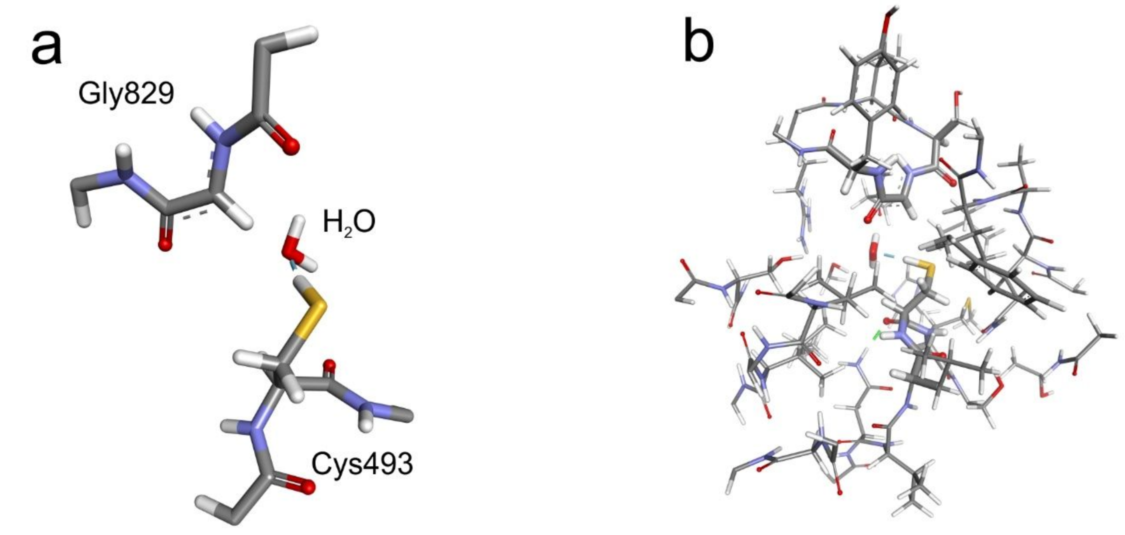

Figure S2. The QM (HL) part of the QM:MM BSS apoenzyme models used in the study of H_2O-assisted radical transfer. S-HL for a) S-QM and b) B-HL.

Results

Fumarate binding pocket

top view side view

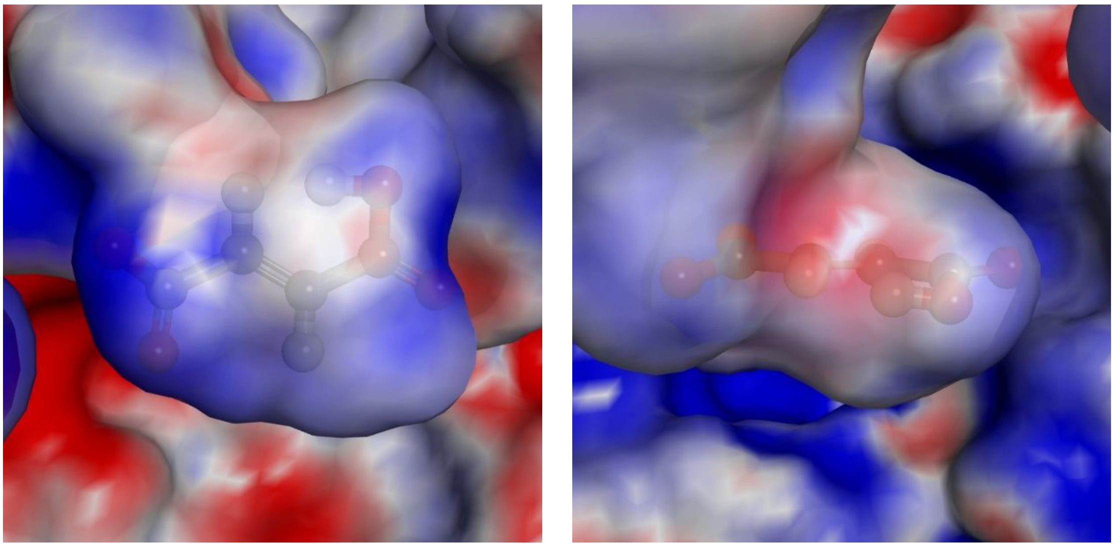

Figure S3. The interpolated charge of the BSS protein depicted on the surface of the fumarate binding pocket. The blue regions indicate a positive charge while the red regions indicate a negative charge. The left blue region on the top view originates from Arg508 while the right blue region from the main chain of the Met404 and Cys494. The negative charge patch visible in the side view is located close to the protonated carboxyl group and comes from the carbonyl group of Asn615.

MD simulations

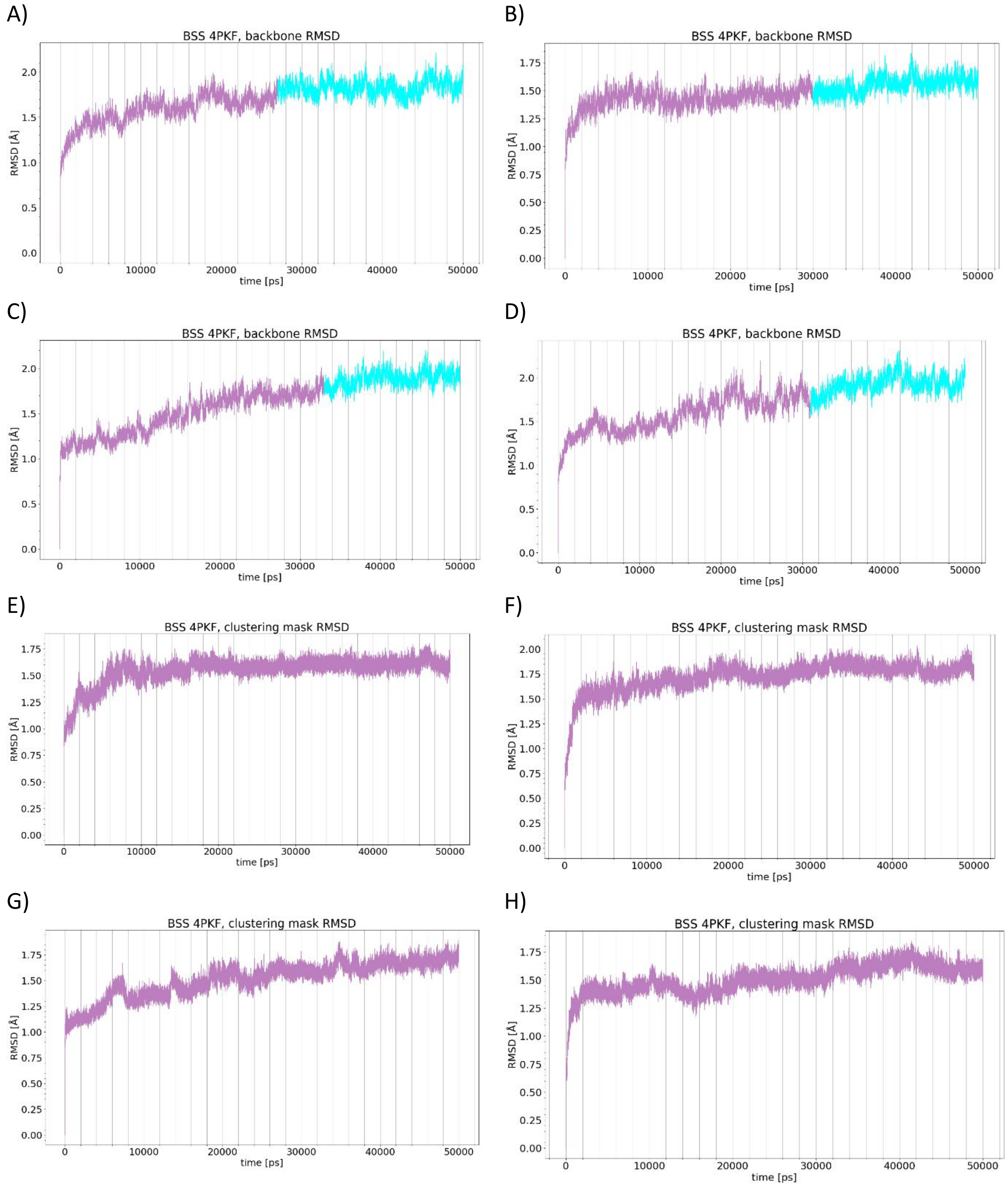

Figure S4. RMSD of the MD simulations conducted for apoenzyme A-D) RMSD of the main chain heavy atoms with trajectory sections taken for clustering analysis (blue) E-F) RMSD of the clustering mask of residues composing the active site.

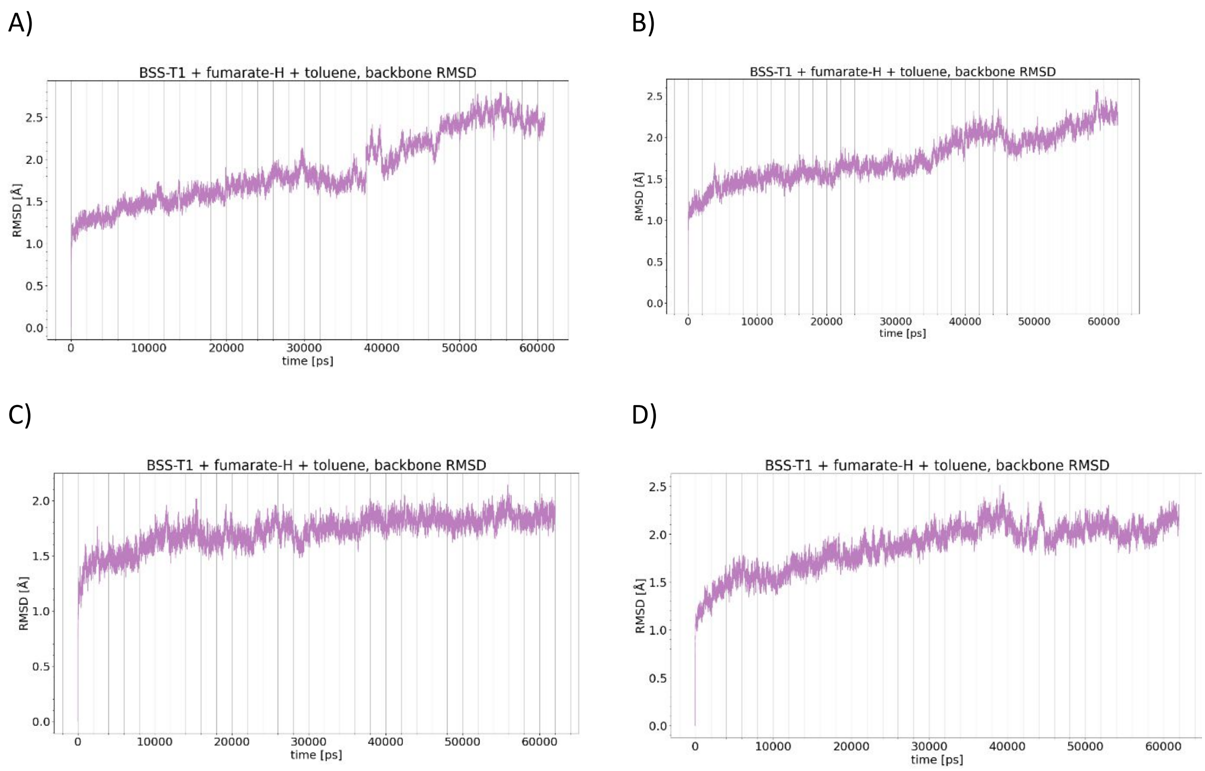

Figure S5. RMSD of four MD simulations conducted for holoenzyme A-D) RMSD of the mainchain heavy atoms; The structure of the model used in QMMM was taken from the simulation depicted in panel A from the frame at 24973 ps, The statistical analysis was conducted for 10-61 ms of sim 1, 10-34 ns of sim 2, 16-62 ns of sim 3 and 40-62 ns of sim 4.

A)

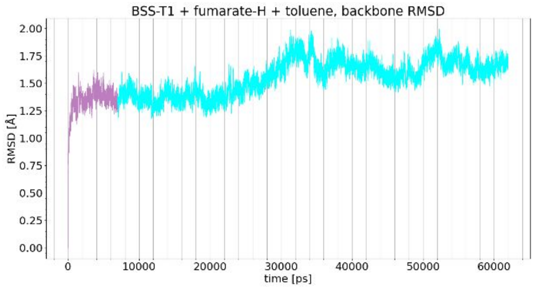

B)

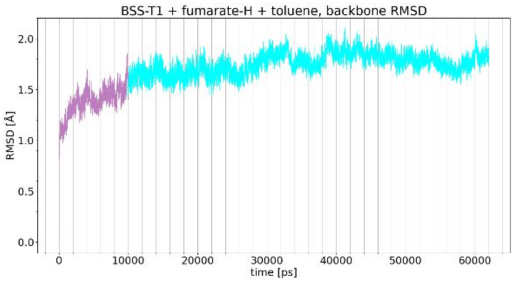

C)

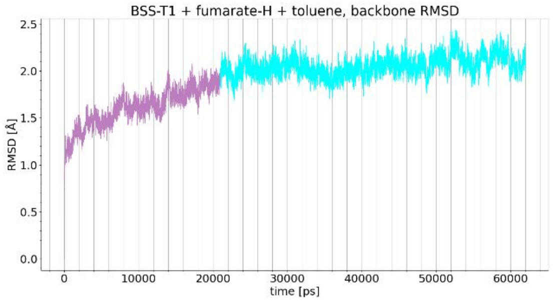

D)

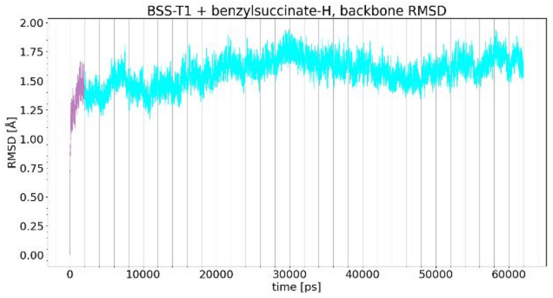

E)

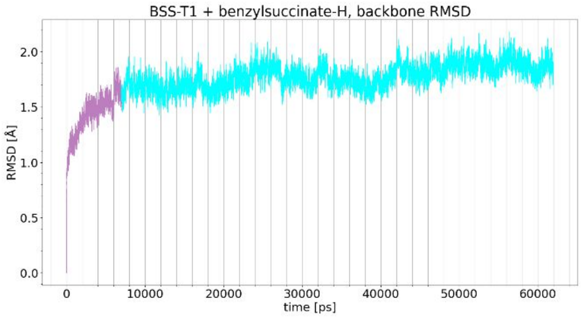

F)

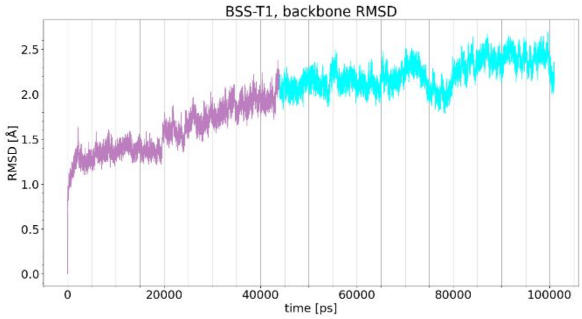

H)

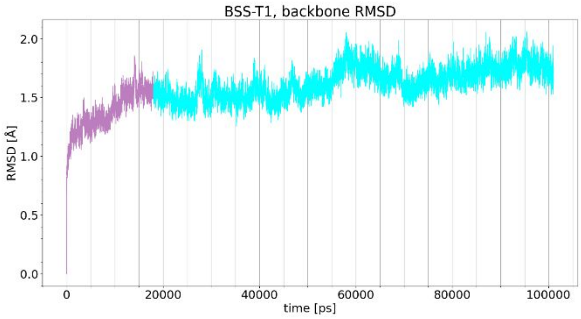

Figure S6. RMSD of the mainchain heavy atoms for the MD simulations conducted with radical Cys for A-C) toluene and monoprotonated fumarate, D-E) monoprotonated R-benzylsuccinate, G-H) apoenzyme; sections of trajectories in blue were taken for statistical analysis,

A) E:S with radical Gly

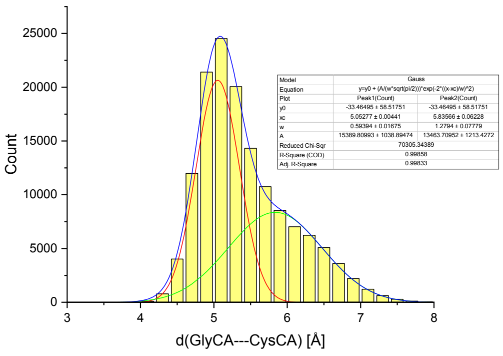

B) apo with radical Gly

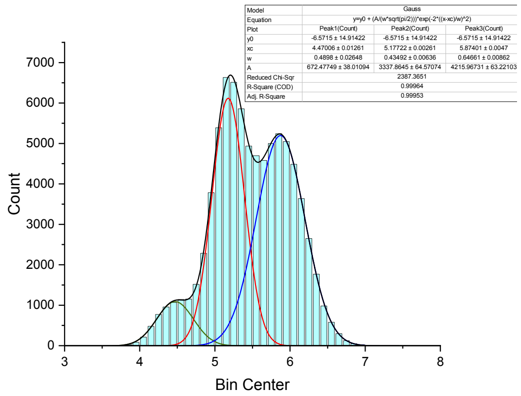

C) E:S with radical Cys

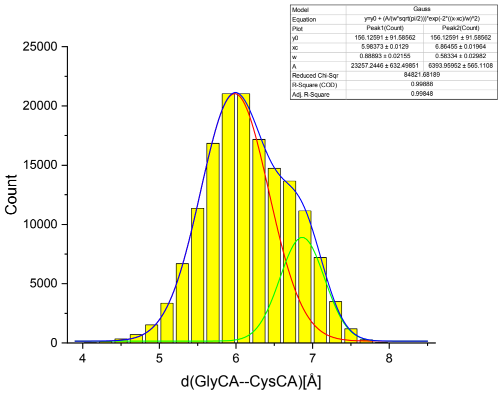

D) apo with radical Cys

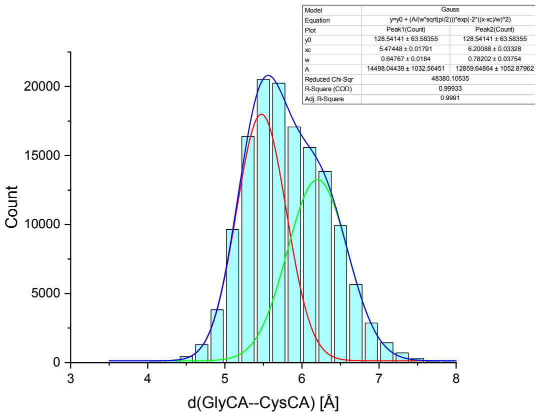

Figure S7. Distribution of distances Gly928Cα—Cα-Cys493 in MD simulations with radical Gly of A) E:S model, B) apo model; with radical Cys of C) E:S model, D) apo model

QM:MM calculations

Table S5 Electronic energies of the holoenzyme calculated for small and big high layers (S-QM and B-QM) and vibrational corrections calculated for 303K, 1 atm. and scaling factor of 0.9806; S-QM dzvp - B3LYP/6-31g(d,p):AMBER; S-QM tzvp B3LYP/6-311g+(2d,2p); B-QM dzvp B3LYP/6-31g(d,p)/D3; B-QM tzvp B3LYP/6-311g+(2d,2p)/D3; corrections: ZPE – zero point energy, Thermal E – thermal energy, H – enthalpy, G – Gibbs free energy.

Substrate-bound proR (*re* attack)									
		E S-QM dzvp [Ha]	E S-QM tzvp [Ha]	E B-QM dzvp [Ha]	E B-QM tzvp [Ha]	ZPE [Ha]	Thermal E [Ha]	H [Ha]	G [Ha]
***re* H GlyH**	E:S	-2365.557847	-2366.209618	-8121.689122	-8123.912201	24.060562	25.647039	25.647999	22.489731
	TS^{ES}	-2365.557847	-2366.190907	-8121.670536	-8123.894122	24.059419	25.644356	25.645315	22.490913
	I^{ES}	-2365.557847	-2366.233018	-8121.707772	-8123.928449	24.065249	25.651198	25.652157	22.494700
***re* D GlyH**	E:S	-2365.557847	-2366.209618	-8121.689122	-8123.912201	24.058079	25.644770	25.645729	22.487049
	TS^{ES}	-2365.537950	-2366.190907	-8121.670536	-8123.894122	24.058008	25.643143	25.644103	22.489397
	I^{ES}	-2365.581936	-2366.233018	-8121.707772	-8123.928449	24.061921	25.648005	25.648964	22.491263
***re* H GlyD**	E:S	-2365.557847	-2366.209618	-8121.689122	-8123.912201	24.057542	25.644212	25.645172	22.486540
	TS^{ES}	-2365.537950	-2366.190907	-8121.670536	-8123.894122	24.056193	25.641289	25.642248	22.487592
	I^{ES}	-2365.581936	-2366.233018	-8121.707772	-8123.928449	24.061938	25.648026	25.648985	22.491280
***re* D GlyD**	E:S	-2365.557847	-2366.209618	-8121.689122	-8123.912201	24.055059	25.641943	25.642902	22.483858
	TS^{ES}	-2365.537950	-2366.190907	-8121.670536	-8123.894122	24.054783	25.640079	25.641038	22.486078
	I^{ES}	-2365.581936	-2366.233018	-8121.707772	-8123.928449	24.058587	25.644803	25.645762	22.487828

Substrate-bound proS									
		E S-QM dzvp [Ha]	E S-QM tzvp [Ha]	E B-QM dzvp [Ha]	E B-QM tzvp [Ha]	ZPE [kJ/mol]	Thermal E [kJ/mol]	H [kJ/mol]	G [kJ/mol]
***si* H GlyH**	E:S	-2365.555280	-2366.206907	-8121.700046	-8123.915361	24.065979	25.650817	25.651777	22.499001
	TS^{ES}	-2365.525701	-2366.177298	-8121.674079	-8123.887098	24.062325	25.646227	25.647187	22.497488
	I^{ES}	-2365.580888	-2366.230491	-8121.720583	-8123.932370	24.070954	25.654993	25.655953	22.504854
***si* D GlyH**	E:S	-2365.555280	-2366.206907	-8121.700046	-8123.915361	24.063549	25.648597	25.649556	22.496326
	TS^{ES}	-2365.525701	-2366.177298	-8121.674079	-8123.887098	24.060954	25.645025	25.645984	22.496018
	I^{ES}	-2365.580888	-2366.230491	-8121.720583	-8123.932370	24.067648	25.651826	25.652785	22.501437
***si* H GlyD**	E:S	-2365.555280	-2366.206907	-8121.700046	-8123.915361	24.062954	25.647984	25.648944	22.495806
	TS^{ES}	-2365.525701	-2366.177298	-8121.674079	-8123.887098	24.059083	25.643141	25.644101	22.494148
	I^{ES}	-2365.580888	-2366.230491	-8121.720583	-8123.932370	24.067636	25.651811	25.652770	22.501424
***si* D GlyD**	E:S	-2365.555280	-2366.206907	-8121.700046	-8123.915361	24.060523	25.645763	25.646723	22.493130
	TS^{ES}	-2365.525701	-2366.177298	-8121.674079	-8123.887098	24.057718	25.641945	25.642905	22.492685
	I^{ES}	-2365.580888	-2366.230491	-8121.720583	-8123.932370	24.064307	25.648613	25.649573	22.497994
Product-bound proR (*re* attack)									
		E S-QM dzvp [Ha]	E S-QM tzvp [Ha]	E B-QM dzvp [Ha]	E B-QM tzvp [Ha]	ZPE [kJ/mol]	Thermal E [kJ/mol]	H [kJ/mol]	G [kJ/mol]
***re* H GlyH**	E:S	-2365.523846	-2366.155622	-8121.673915	-8123.871721	24.082470	25.660248	25.661208	22.531462
	TS^{ES}	-2365.478142	-2366.111094	-8121.638286	-8123.837132	24.076352	25.653280	25.654239	22.527184
	I^{ES}	-2365.497373	-2366.131963	-8121.655215	-8123.855597	24.078949	25.656978	25.657937	22.527532
***re* D GlyH**	E:S	-2365.523846	-2366.155622	-8121.673915	-8123.871721	24.079153	25.657068	25.658027	22.528034
	TS^{ES}	-2365.478142	-2366.111094	-8121.638286	-8123.837132	24.074927	25.652056	25.653015	22.525654

	I^{ES}	-2365.497373	-2366.131963	-8121.655215	-8123.855597	24.076487	25.654732	25.655691	22.524843
re **H GlyD**	E:S	-2365.523846	-2366.155622	-8121.673915	-8123.871721	24.079165	25.657082	25.658042	22.528046
	TS^{ES}	-2365.478142	-2366.111094	-8121.638286	-8123.837132	24.073113	25.650198	25.651158	22.523851
	I^{ES}	-2365.497373	-2366.131963	-8121.655215	-8123.855597	24.075930	25.654152	25.655111	22.524340
re **D GlyD**	E:S	-2365.523846	-2366.155622	-8121.673915	-8123.871721	24.075826	25.653871	25.654831	22.524604
	TS^{ES}	-2365.478142	-2366.111094	-8121.638286	-8123.837132	24.071688	25.648976	25.649935	22.522322
	I^{ES}	-2365.497373	-2366.131963	-8121.655215	-8123.855597	24.073468	25.651906	25.652865	22.521651
**Product-bound proS (*si* attack)**									
		E S-QM dzvp [Ha]	E S-QM tzvp [Ha]	E B-QM dzvp [Ha]	E B-QM tzvp [Ha]	ZPE [kJ/mol]	Thermal E [kJ/mol]	H [kJ/mol]	G [kJ/mol]
si **H GlyH**	E:S	-2365.523952	-2366.155659	-8121.674164	-8123.871739	24.082444	25.660234	25.661194	22.531535
	TS^{ES}	-2365.471726	-2366.105126	-8121.627560	-8123.826681	24.074338	25.651880	25.652839	22.524138
	I^{ES}	-2365.498135	-2366.132756	-8121.654462	-8123.855133	24.078656	25.656743	25.657703	22.527248
si **D GlyH**	E:S	-2365.523952	-2366.155659	-8121.674164	-8123.871739	24.082444	25.660234	25.661194	22.531535
	TS^{ES}	-2365.471726	-2366.105126	-8121.627560	-8123.826681	24.074338	25.651880	25.652839	22.524138
	I^{ES}	-2365.498135	-2366.132756	-8121.654462	-8123.855133	24.078656	25.656743	25.657703	22.527248
si **H GlyD**	E:S	-2365.523952	-2366.155659	-8121.674164	-8123.871739	24.079124	25.657049	25.658009	22.528104
	TS^{ES}	-2365.471726	-2366.105126	-8121.627560	-8123.826681	24.071095	25.648794	25.649753	22.520796
	I^{ES}	-2365.498135	-2366.132756	-8121.654462	-8123.855133	24.075645	25.653925	25.654884	22.524062
si **D GlyD**	E:S	-2365.523952	-2366.155659	-8121.674164	-8123.871739	24.075797	25.653854	25.654814	22.524675
	TS^{ES}	-2365.471726	-2366.105126	-8121.627560	-8123.826681	24.071095	25.648794	25.649753	22.520796
	I^{ES}	-2365.498135	-2366.132756	-8121.654462	-8123.855133	24.073194	25.651687	25.652647	22.521377

Table S6. Differences in electronic energies corrected by thermal energy calculated for the holoenzyme at different levels of theory.

			Δ(E+thermal) S-QM dzvp [kJ/mol]	Δ(E+thermal) S-QM tzvp [kJ/mol]	Δ(E+thermal) B-QM dzvp [kJ/mol]	Δ(E+thermal) B-QM tzvp [kJ/mol]
E:S *re* attack	*re* H GlyH	E:S	0.00	0.00	0.00	0.00
		TS^{ES}	45.20	42.10	41.80	40.40
		I^{ES}	-52.30	-50.50	-38.00	-31.70
	re D GlyH	E:S	0.00	0.00	0.00	0.00
		TS^{ES}	48.00	44.90	44.50	43.20
		I^{ES}	-54.80	-52.90	-40.50	-34.20
	re H GlyD	E:S	0.00	0.00	0.00	0.00
		TS^{ES}	43.10	40.00	39.70	39.80
		I^{ES}	-54.70	-52.90	-40.40	-32.60
	re D GlyD	E:S	0.00	0.00	0.00	0.00
		TS^{ES}	47.30	44.20	43.90	42.60
		I^{ES}	-55.70	-53.90	-41.50	-35.20
			Δ(E+thermal) S-QM dzvp [kJ/mol]	Δ(E+thermal) S-QM tzvp [kJ/mol]	Δ(E+thermal) B-QM dzvp [kJ/mol]	Δ(E+thermal) B-QM tzvp [kJ/mol]
E:S *si* attack	*si* H GlyH	E:S	0.00	0.00	0.00	0.00
		TS^{ES}	65.60	65.70	56.10	62.20
		I^{ES}	-56.30	-51.00	-43.00	-33.70
	si D GlyH	E:S	0.00	0.00	0.00	0.00
		TS^{ES}	68.30	68.40	58.80	64.80
		I^{ES}	-58.80	-53.40	-45.40	-36.20

	si H GlyD	E:S	0.00	0.00	0.00	0.00
		TS^{ES}	64.90	65.00	55.50	61.50
		I^{ES}	-57.20	-51.90	-43.90	-34.60
	si D GlyD	E:S	0.00	0.00	0.00	0.00
		TS^{ES}	67.60	67.70	58.20	64.20
		I^{ES}	-59.80	-54.40	-46.40	-37.20
			Δ(E+thermal) S-QM dzvp [kJ/mol]	Δ(E+thermal) S-QM tzvp [kJ/mol]	Δ(E+thermal) B-QM dzvp [kJ/mol]	Δ(E+thermal) B-QM tzvp [kJ/mol]
E:P *re* attack	*re* H GlyH	E:S	0	0.00	0.00	0.00
		TS^{ES}	101.7	98.60	75.30	72.50
		I^{ES}	60.9	53.50	40.50	33.70
	re D GlyH	E:S	0	0.00	0.00	0.00
		TS^{ES}	106.8	103.80	80.40	77.70
		I^{ES}	63.4	56.00	43.00	36.20
	re H GlyD	E:S	0	0.00	0.00	0.00
		TS^{ES}	101.9	98.80	75.50	72.70
		I^{ES}	61.8	54.40	41.40	34.60
	re D GlyD	E:S	0	0.00	0.00	0.00
		TS^{ES}	107.1	104.10	80.70	78.00
		I^{ES}	64.3	57.00	43.90	37.20
			Δ(E+thermal) S-QM dzvp [kJ/mol]	Δ(E+thermal) S-QM tzvp [kJ/mol]	Δ(E+thermal) B-QM dzvp [kJ/mol]	Δ(E+thermal) B-QM tzvp [kJ/mol]
E:P *si* attack	*si* H GlyH	E:S	0	0.00	0.00	0.00
		TS^{ES}	115.2	110.70	100.40	96.40
		I^{ES}	58.6	51.00	42.60	34.40

	si D GlyH	E:S	0	0.00	0.00	0.00
		TS^{ES}	115.2	110.70	100.40	104.70
		I^{ES}	58.6	51.00	42.60	36.90
	si H GlyD	E:S	0	0.00	0.00	0.00
		TS^{ES}	115.4	111.00	100.70	96.60
		I^{ES}	59.6	51.90	43.50	35.40
	si D GlyD	E:S	0	0.00	0.00	0.00
		TS^{ES}	123.8	119.40	109.10	105.00
		I^{ES}	62.1	54.40	46.00	37.90

Table S7. Electronic energies of the apoenzyme calculated for small and big high layer (S-QM and B-QM) and vibrational corrections calculated for 303K, 1 atm. and scaling factor of 0.9806; S-QM dzvp - B3LYP/6-31g(d,p):AMBER; S-QM tzvp B3LYP/6-311g+(2d,2p); B-QM dzvp B3LYP/6-31g(d,p)/D3; B-QM tzvp B3LYP/6-311g+(2d,2p)/D3; corrections: ZPE – zero point energy, Thermal E – thermal energy, H – enthalpy, G – Gibbs free energy.

**Apoenzyme *re* attack**									
		**E S-QM dzvp [Ha]**	**E S-QM tzvp [Ha]**	**E B-QM dzvp [Ha]**	**E B-QM tzvp [Ha]**	**ZPE [Ha]**	**Thermal E [Ha]**	**H [Ha]**	**G [Ha]**
***re* H GlyH**	**E**	-1388.416787	-1388.746900	-9894.194062	-9896.932831	23.955326	25.514187	25.515147	22.423756
	**TS^{apo}**	-1388.378023	-1388.702496	-9894.157183	-9896.889135	23.954017	25.511245	25.512205	22.425430
	**I**	-1388.427289	-1388.755364	-9894.198947	-9896.932550	23.957761	25.516637	25.517596	22.424543
***re* D GlyH**	**E**	-1388.416787	-1388.746900	-9894.194062	-9896.932831	23.952768	25.511847	25.512807	22.421012
	**TS^{apo}**	-1388.378023	-1388.702496	-9894.157183	-9896.889135	23.952582	25.509967	25.510927	22.423918
	**I**	-1388.427289	-1388.755364	-9894.198947	-9896.932550	23.954385	25.513387	25.514346	22.421066
***re* H GlyD**	**E**	-1388.416787	-1388.746900	-9894.194062	-9896.932831	23.952220	25.511273	25.512233	22.420511
	**TS^{apo}**	-1388.378023	-1388.702496	-9894.157183	-9896.889135	23.950645	25.508015	25.508975	22.421982
	**I**	-1388.427289	-1388.755364	-9894.198947	-9896.932550	23.954457	25.513473	25.514432	22.421132
***re* D GlyD**	**E**	-1388.416787	-1388.746900	-9894.194062	-9896.932831	23.949662	25.508933	25.509893	22.417767
	**TS^{apo}**	-1388.378023	-1388.702496	-9894.157183	-9896.889135	23.949216	25.506742	25.507702	22.420477
	**I**	-1388.427289	-1388.755364	-9894.198947	-9896.932550	23.951060	25.510195	25.511155	22.417641

**Apoenzyme *si* attack**									
		**E S-QM dzvp [Ha]**	**E S-QM tzvp [Ha]**	**E B-QM dzvp [Ha]**	**E B-QM tzvp [Ha]**	**ZPE [Ha]**	**Thermal E [Ha]**	**H [Ha]**	**G [Ha]**
***si* H GlyH**	**E**	-1388.373675	-1388.704177	-9894.157596	-9896.896266	23.950771	25.510693	25.511653	22.419566
	**TS^{apo}**	-1388.351292	-1388.678170	-9894.129976	-9896.864073	23.950368	25.509189	25.510149	22.415631
	**I**	-1388.369728	-1388.697719	-9894.137935	nd*	23.95836	25.516276	25.517236	22.431036
***si* D GlyH**	**E**	-1388.373675	-1388.704177	-9894.157596	-9896.896266	23.9476	25.507699	25.508658	22.416268
	**TS^{apo}**	-1388.351292	-1388.678170	-9894.129976	-9896.864073	23.947055	25.506023	25.506983	22.412235
	**I**	-1388.369728	-1388.697719	-9894.137935	nd*	23.955025	25.513078	25.514037	22.427596
	**E**	-1388.373675	-1388.704177	-9894.157596	-9896.896266	23.948319	25.508457	25.509416	22.416877

***si* H GlyD**	**TS^{apo}**	-1388.351292	-1388.678170	-9894.129976	-9896.864073	23.948948	25.507928	25.508887	22.414132
	**I**	-1388.369728	-1388.697719	-9894.137935	nd*	23.955025	25.51307	25.51403	22.427595
***si* D GlyD**	**E**	-1388.373675	-1388.704177	-9894.157596	-9896.896266	23.945148	25.505463	25.506422	22.413579
	**TS^{apo}**	-1388.351292	-1388.678170	-9894.129976	-9896.864073	23.945638	25.504764	25.505723	22.410740
	**I**	-1388.369728	-1388.697719	-9894.137935	nd*	23.95167	25.509844	25.510804	22.424142

* the lacking electronic energy of I^{ES} at **E B-QM tzvp level of theory** was estimated based on an energy difference between TS^{apo} and I at E B-QM dzvp level

Table S8. Differences in electronic energies corrected by thermal energy calculated for the apoenzyme at different levels of theory

		**H transfer from Cys to Gly^{rad}**					**H transfer from Gly to Cys^{rad}**			
		**Δ(E+thermal) S-QM dzvp [kJ/mol]**					**Δ(E+thermal) S-QM dzvp [kJ/mol]**			
		re H GlyH	*re* D GlyH	*re* H GlyD	*re* D GlyD		*R*-H $GlyH_2$	*R*-D *R*-GlyHD	*R*-H *S*-GlyDH	*R*-D *R*-$GlyD_2$
re/proR	**E**	0.0	0.0	0.0	0.0	**I**	0.0	0.0	0.0	0.0
	**TS^{apo}**	94.1	96.8	93.2	96.0	**TS^{apo}**	115.2	120.4	115.0	120.3
	**I**	-21.1	-23.5	-21.8	-24.3	**E**	21.1	23.5	21.8	24.3
		si H GlyH	*si* D GlyH	*si* H GlyD	*si* D GlyD		*S*-H $GlyH_2$	*S*-D *S*-GlyDH	*S*-H *R*-GlyHD	*S*-D *R*-$GlyD_2$
si/proS	**E**	0.0	0.0	0.0	0.0	**I**	0.0	0.0	0.0	0.0
	**TS^{apo}**	54.8	54.4	57.4	56.9	**TS^{apo}**	29.8	29.9	34.9	35.1
	**I**	25.0	24.5	22.5	21.9	**E**	-25.0	-24.5	-22.5	-21.9
		**Δ(E+thermal) S-QM tzvp [kJ/mol]**					**Δ(E+thermal) S-QM tzvp [kJ/mol]**			
		re H GlyH	*re* D GlyH	*re* H GlyD	*re* D GlyD		*R*-H $GlyH_2$	*R*-D *R*-GlyHD	*R*-H *S*-GlyDH	*R*-D *R*-$GlyD_2$
re/proR	**E**	0.0	0.0	0.0	0.0	**I**	0.0	0.0	0.0	0.0
	**TS^{apo}**	108.9	111.6	108.0	110.8	**TS^{apo}**	124.7	129.8	124.5	129.7
	**I**	-15.8	-18.2	-16.4	-18.9	**E**	15.8	18.2	16.4	18.9
		si H GlyH	*si* D GlyH	*si* H GlyD	*si* D GlyD		*S*-H $GlyH_2$	*S*-D *S*-GlyDH	*S*-H *R*-GlyHD	*S*-D *R*-$GlyD_2$
si/proS	**E**	0.0	0.0	0.0	0.0	**I**	0.0	0.0	0.0	0.0
	**TS^{apo}**	64.3	63.9	66.9	66.4	**TS^{apo}**	32.7	32.8	37.8	38.0
	**I**	31.6	31.1	29.1	28.5	**E**	-31.6	-31.1	-29.1	-28.5

		Δ(E+thermal) B-QM dzvp [kJ/mol]					Δ(E+thermal) B-QM dzvp [kJ/mol]			
		re H GlyH	*re* D GlyH	*re* H GlyD	*re* D GlyD		*R*-H $GlyH_2$	*R*-D *R*-GlyHD	*R*-H *S*-GlyDH	*R*-D *R*-$GlyD_2$
re/proR	**E**	0.0	0.0	0.0	0.0	**I**	0.0	0.0	0.0	0.0
	**TS^{apo}**	89.1	91.9	88.3	91.1	**TS^{apo}**	95.5	100.7	95.3	100.6
	**I**	-6.4	-8.8	-7.0	-9.5	**E**	6.4	8.8	7.0	9.5
		si H GlyH	*si* D GlyH	*si* H GlyD	*si* D GlyD		*S*-H $GlyH_2$	*S*-D *S*-GlyDH	*S*-H *R*-GlyHD	*S*-D *R*-$GlyD_2$
si/proS	**E**	0.0	0.0	0.0	0.0	**I**	0.0	0.0	0.0	0.0
	**TS^{apo}**	68.6	68.6	68.6	68.6	**TS^{apo}**	2.3	2.4	7.4	7.6
	**I**	66.3	66.3	66.3	66.3	**E**	-66.3	-65.7	-63.7	-63.1

		Δ(E+thermal) B-QM tzvp [kJ/mol]					Δ(E+thermal) B-QM tzvp [kJ/mol]			
		re H GlyH	*re* D GlyH	*re* H GlyD	*re* D GlyD		*R*-H $GlyH_2$	*R*-D *R*-GlyHD	*R*-H *S*-GlyDH	*R*-D *R*-$GlyD_2$
re/proR	**E**	0.0	0.0	0.0	0.0	**I**	0.0	0.0	0.0	0.0
	**TS^{apo}**	107.0	109.8	106.2	109.0	**TS^{apo}**	99.8	105.0	99.7	104.9
	**I**	7.2	4.8	6.5	4.0	**E**	-7.2	-4.8	-6.5	-4.0
		si H GlyH	*si* D GlyH	*si* H GlyD	*si* D GlyD		*S*-H $GlyH_2$	*S*-D *S*-GlyDH	*S*-H *R*-GlyHD	*S*-D *R*-$GlyD_2$
si/proS	**E**	0.0	0.0	0.0	0.0	**I**	0.0*	0.0	0.0	0.0
	**TS^{apo}**	80.6	80.1	83.1	82.7	**TS^{apo}**	2.3	2.4	7.4	7.6
	**I**	78.3*	77.8*	75.7*	75.1*	**E**	-78.3	-77.8	-75.7	-75.1

- Difference in electronic energy between TS^{apo} and I at B-QM tzvp was estimated as the same as between TS^{apo} and I at B-QM dzvp level of theory

Prediction of elementary rate constants

Table S9. Kinetic rate constants and iKIE calculated for E:S complex model

System	transfer	Δ(E+Thermal) [kJ/mol]	k [s^{-1}]	iKIE
E:S *re* attack	*re* H GlyH'	40.4	$6.8*10^{5}$	1.0
	re D GlyH'	43.2	$2.3*10^{5}$	3.01
	re H GlyD'	39.8	$8.7*10^{5}$	0.78
	re D GlyD'	42.6	$2.9*10^{5}$	2.35
E:S *si* attack	*si* H GlyH'	62.2	122	1.0
	si D GlyH'	64.8	42	2.89
	si H GlyD'	61.0	192	0.63
	si D GlyD'	64.2	54	2.24
E:S reverse *R*-transfer	*R*-H $GlyH_2$	72.2	2.3	1.0
	R-D *R*-GlyHD	77.4	0.29	7.87
	R-H *S*-GlyDH	72.4	2.1	1.12
	R-D *R*-$GlyD_2$	77.7	0.25	9.09
E:S reverse *S*-transfer	*S*-H $GlyH_2$	95.8	$1.9*10^{-4}$	1.0
	S-D *S*-GlyDH	101.0	$2.4*10^{-5}$	7.75
	S-H *R*-GlyHD	95.6	$2.1*10^{-4}$	0.91
	S-D *R*-$GlyD_2$	101.4	$2.1*10^{-5}$	8.9

Table S10. Kinetic rate constants and iKIE calculated for E:P complex model

System	transfer	Δ(E+Thermal) [kJ/mol]	k [s^{-1}]	iKIE
E:P *re* attack	*re* H GlyH'	38.8	$1.3*10^{6}$	1.00
	re D GlyH'	41.5	$4.5*10^{5}$	2.90
	re H GlyD'	38.1	$1.7*10^{6}$	0.77
	re D GlyD'	40.8	$5.9*10^{5}$	2.23
E:P *si* attack	*si* H GlyH'	61.9	133	1.00
	si D GlyH'	67.8	13	10.29
	si H GlyD'	61.2	176	0.76
	si D GlyD'	67.1	17	7.79
E:P reverse R-transfer	*R*-H $GlyH_2$	72.5	2	1.00
	R-D *R*-GlyHD	77.7	0.26	7.68
	R-H *S*-GlyDH	72.7	1.8	1.09
	R-D $GlyD_2$	78.0	0.23	8.67
E:P reverse S-transfer	*S*-H $GlyH_2$	96.4	$1.5*10^{-4}$	1.00
	S-D *S*-GlyDH	104.7	$5.7*10^{-6}$	27.07
	S-H *R*-GlyHD	96.6	$1.4*10^{-4}$	1.11
	S-D $GlyD_2$	105.0	$5.0*10^{-6}$	30.97

Table S11. Kinetic rate constants and iKIE calculated for apoenzyme, Δ(E+Thermal)‘ takes into account the energy difference between proR and proS conformations,

**System**	**transfer**	**Δ(E+Thermal) [kJ/mol]**	k [s^{-1}]	**Δ(E+Thermal)‘ [kJ/mol]**	k‘ [s^{-1}]	iKIE
APO *re* attack	*re* H GlyH′	107.0	2.27*10^{-6}	107.0	2.27*10^{-6}	1.00
	re D GlyH′	109.8	7.49*10^{-7}	109.8	7.49*10^{-7}	3.02
	re H GlyD′	106.2	3.15*10^{-6}	106.2	3.15*10^{-6}	0.72
	re D GlyD′	109.0	1.04*10^{-6}	109.0	1.04*10^{-6}	2.19
APO *si* attack	*si* H GlyH′	80.6	8.13*10^{-2}	167.4	8.76*10^{-17}	1.00
	si D GlyH′	80.1	9.73*10^{-2}	166.9	1.05*10^{-16}	0.84
	si H GlyD′	83.1	2.94*10^{-2}	170.0	3.17*10^{-17}	2.76
	si D GlyD′	82.7	3.52*10^{-2}	169.5	3.79*10^{-17}	2.31
APO reverse R-transfer	*R*-H $GlyH_2$	99.8	3.90*10^{-5}	99.8	3.90*10^{-5}	1.00
	R-D *R*-GlyHD	105.0	4.99*10^{-6}	105.0	4.99*10^{-6}	7.81
	R-H *S*-GlyDH	99.7	4.18*10^{-5}	99.7	4.18*10^{-5}	0.93
	R-D $GlyD_2$	104.9	5.17*10^{-6}	104.9	5.17*10^{-6}	7.54
APO reverse S-transfer	*S*-H $GlyH_2$	2.3	2.55*10^{12}	89.1	2.74*10^{-3}	1.00
	S-D GlyDH	2.4	2.46*10^{12}	89.2	2.65*10^{-3}	1.03

The geometries of the stationary points

proR E:S

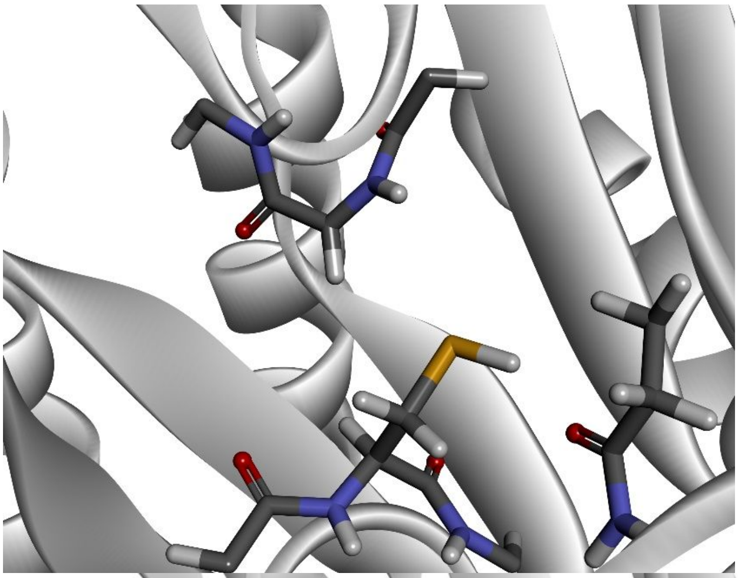

proR TS^{ES}

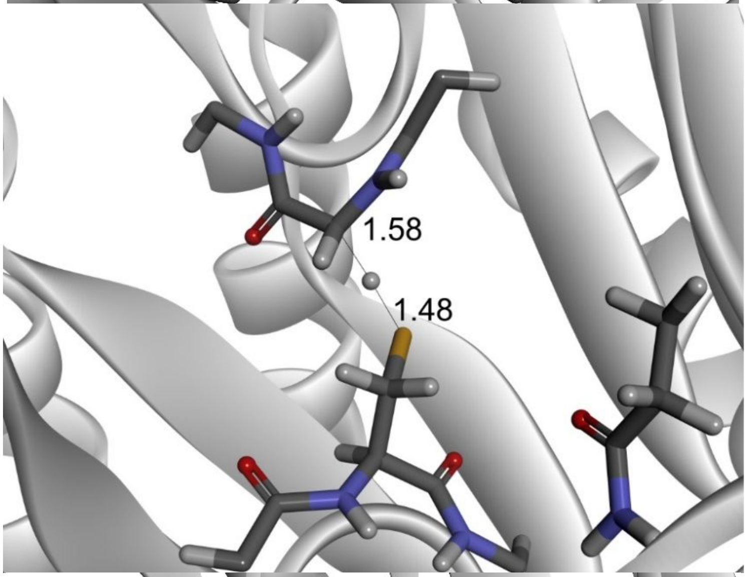

proR I^{ES}

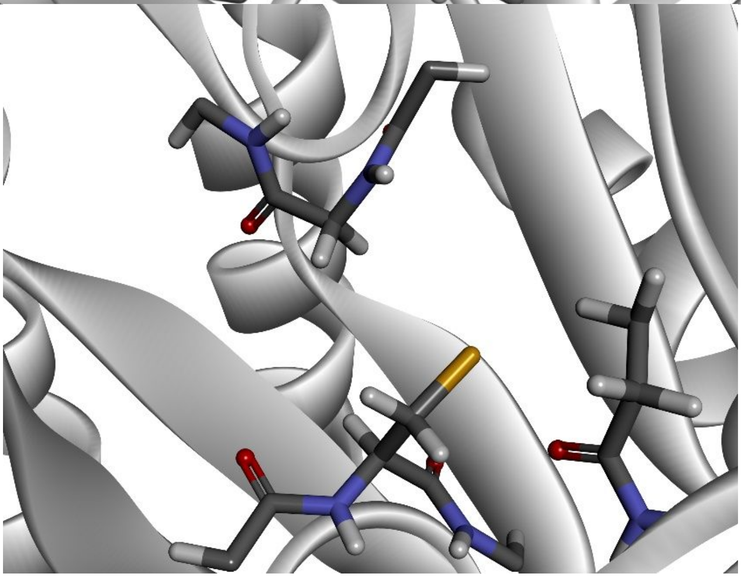

proS E:S

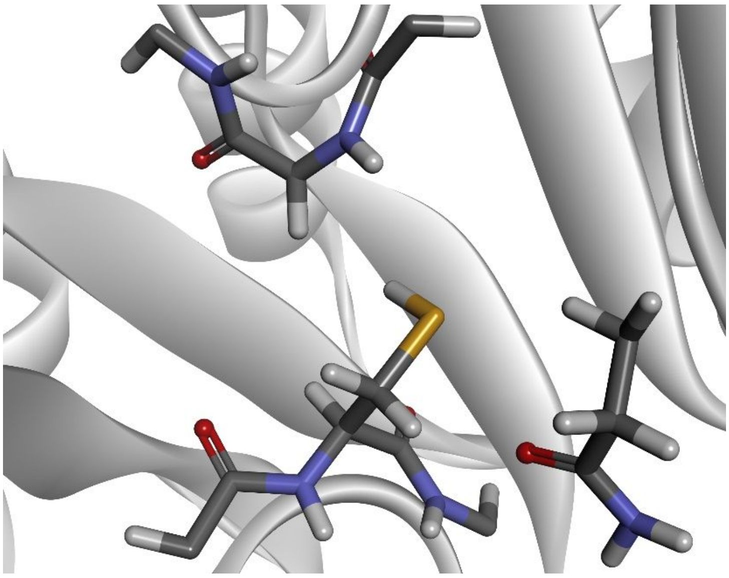

proS TS^{ES}

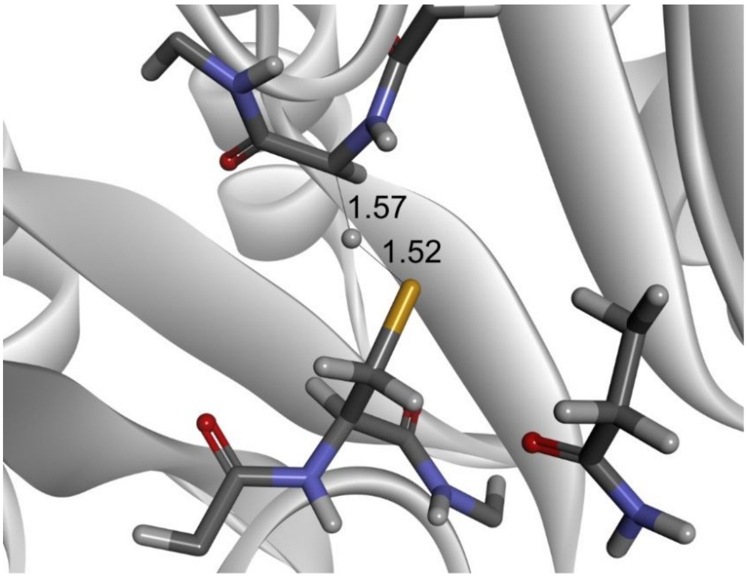

proS I^{ES}

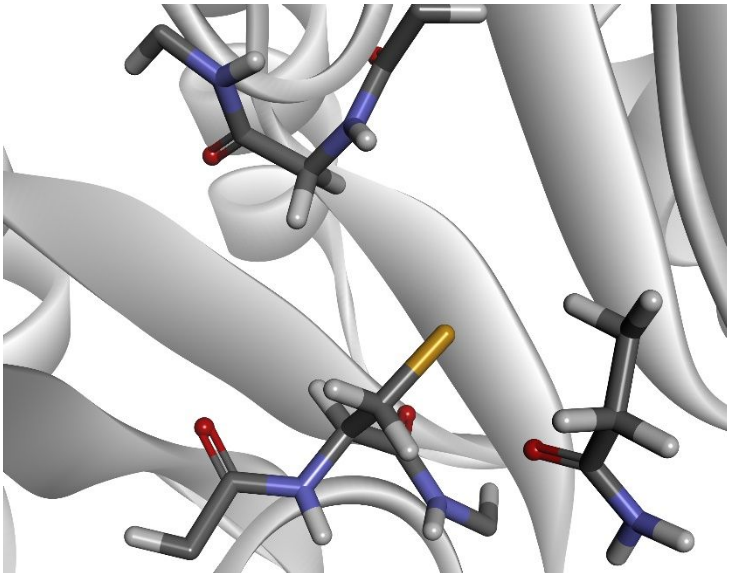

Figure S8. The geometry of stationary points obtained for E:S holoenzyme – *re* and *si* attack.

proR E:P

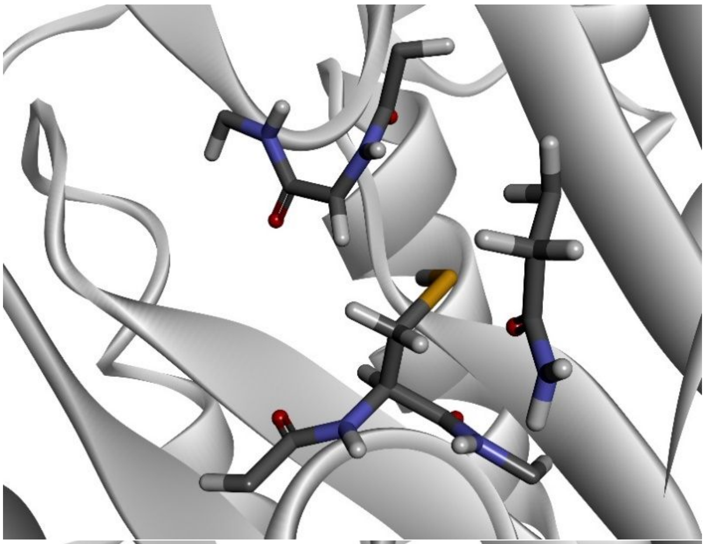

proR TS^{EP}

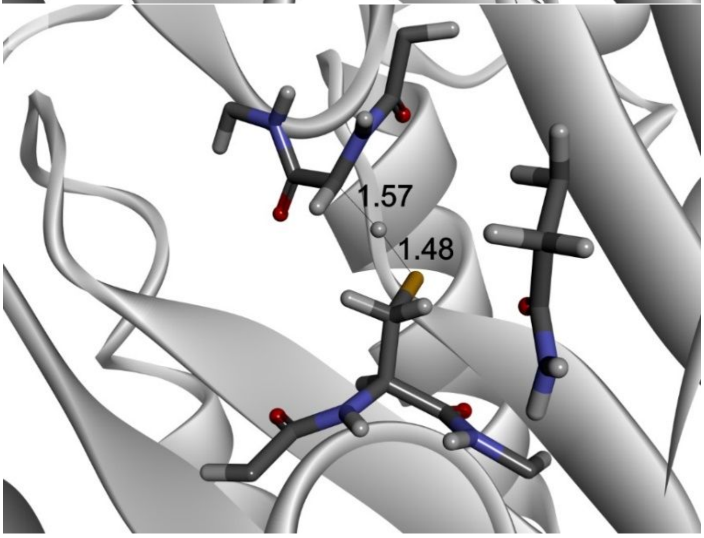

proR I^{EP}

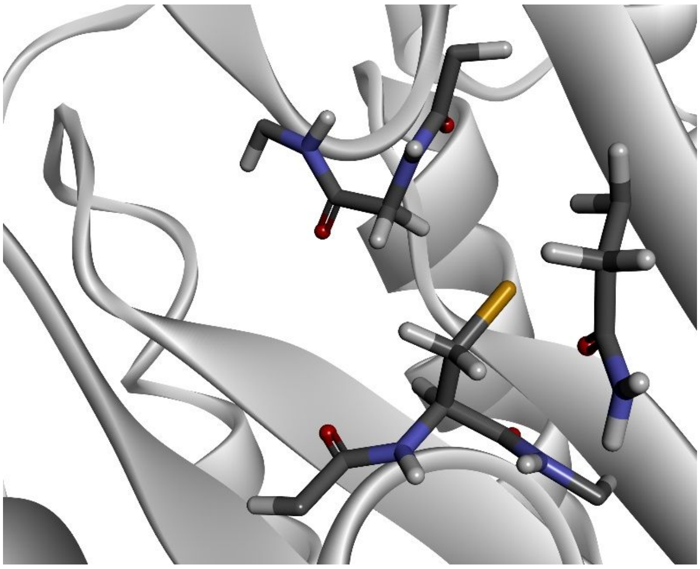

proS E:P

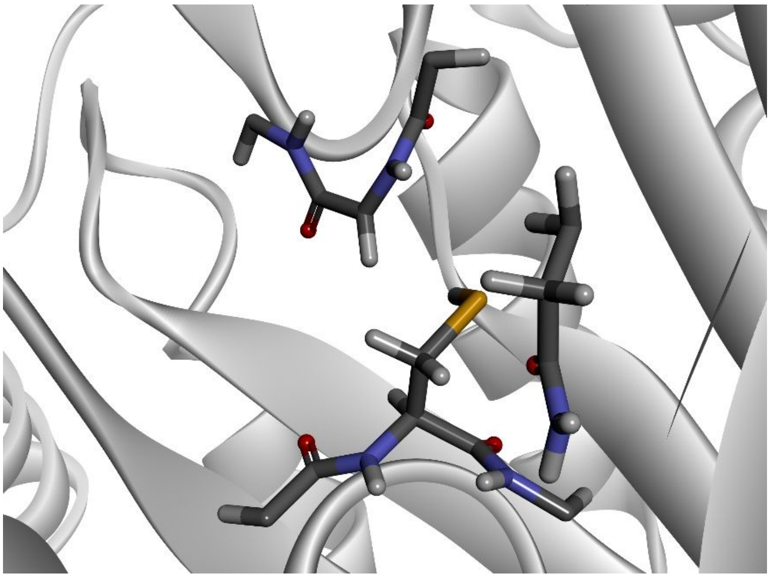

proS TS^{EP}

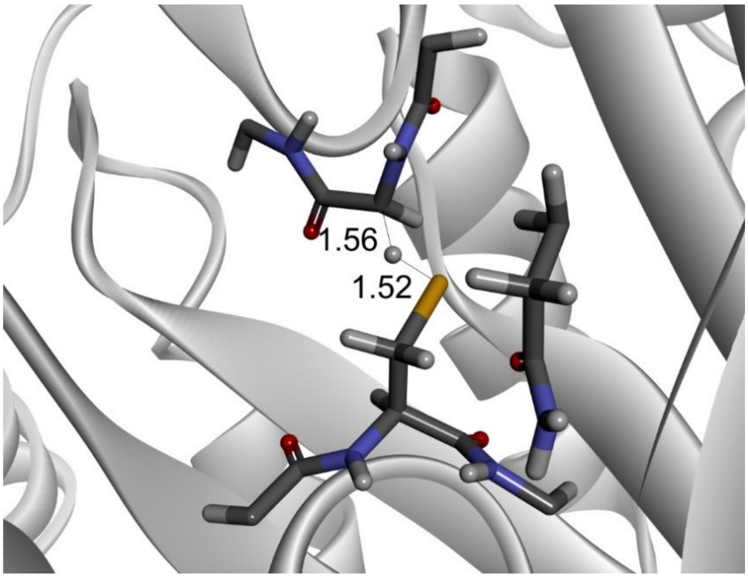

proS I^{EP}

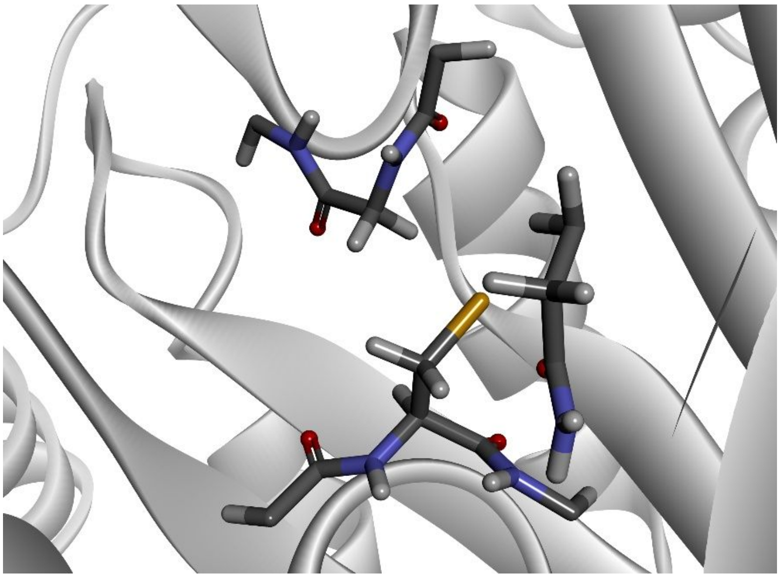

Figure S9. Geometry of stationary points obtained for E:P holoenzyme – *re* and *si* attack.

proR E^{apo}

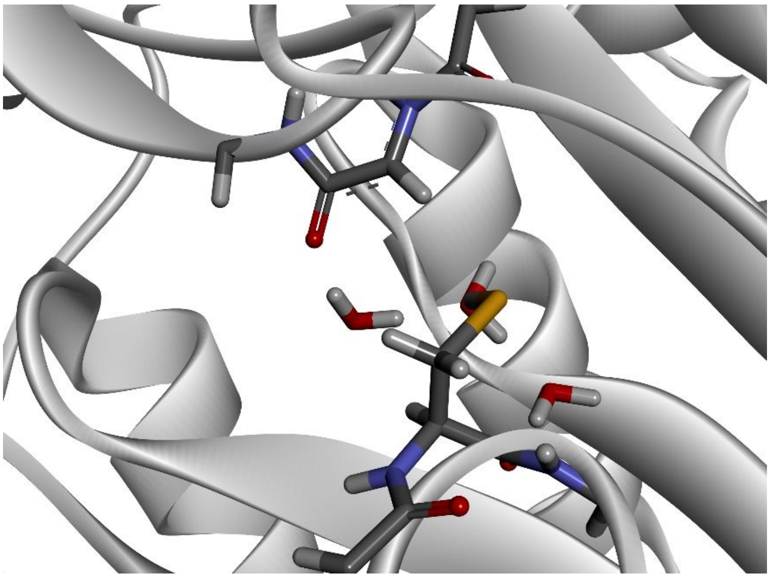

proR TS^{apo}

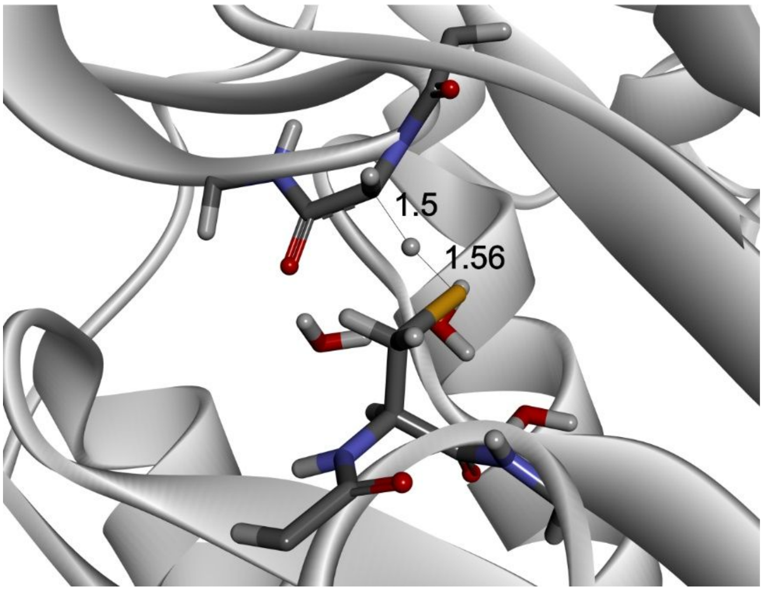

proR I^{apo}

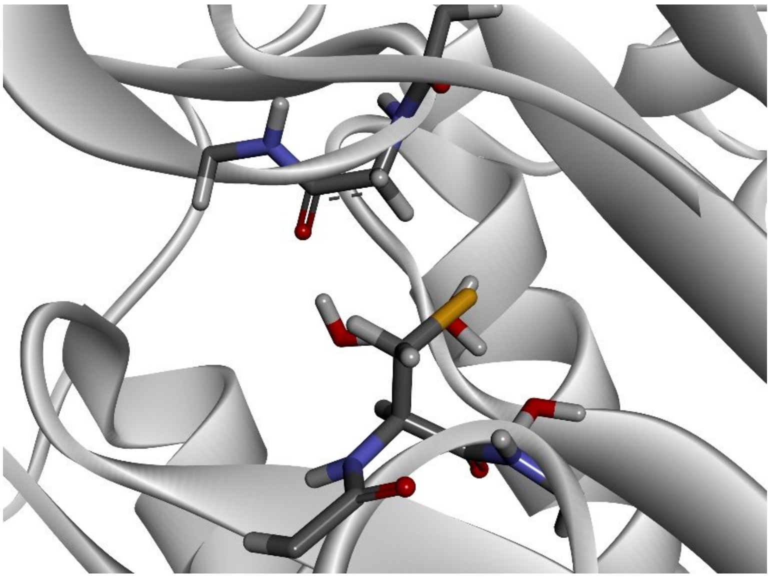

proS E^{apo}

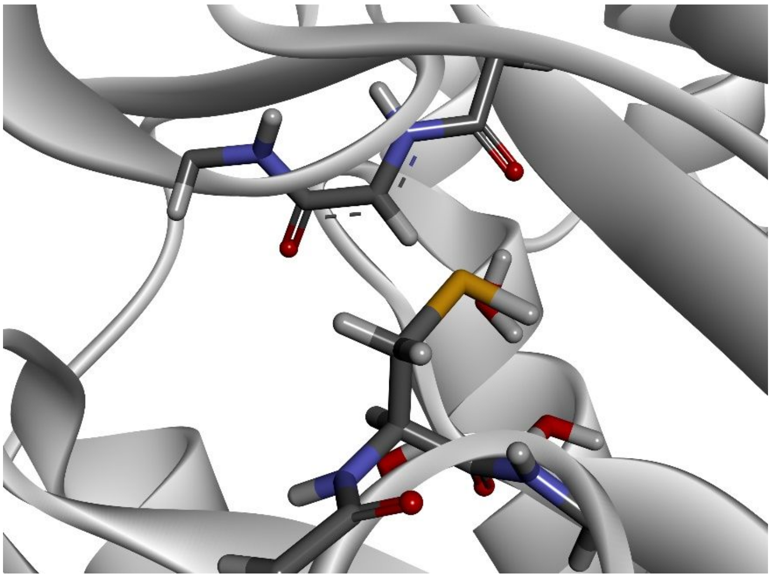

proS TS^{apo}

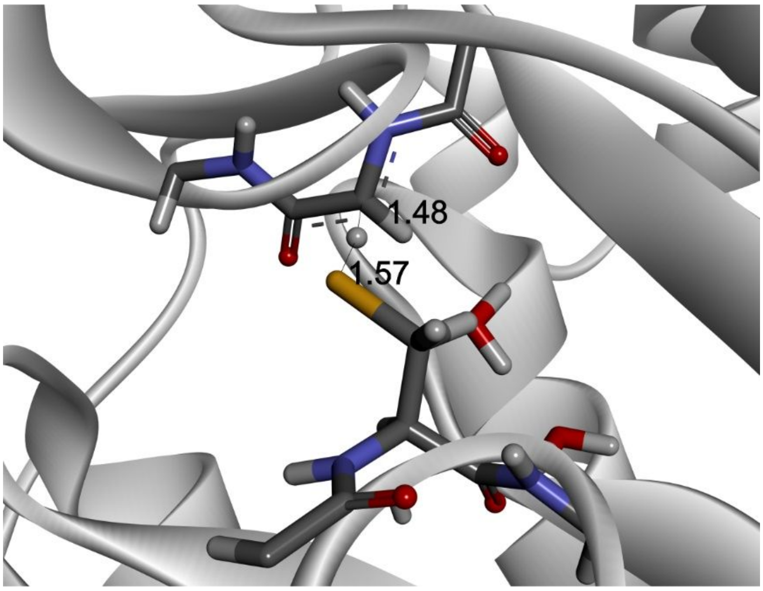

proS I^{apo}

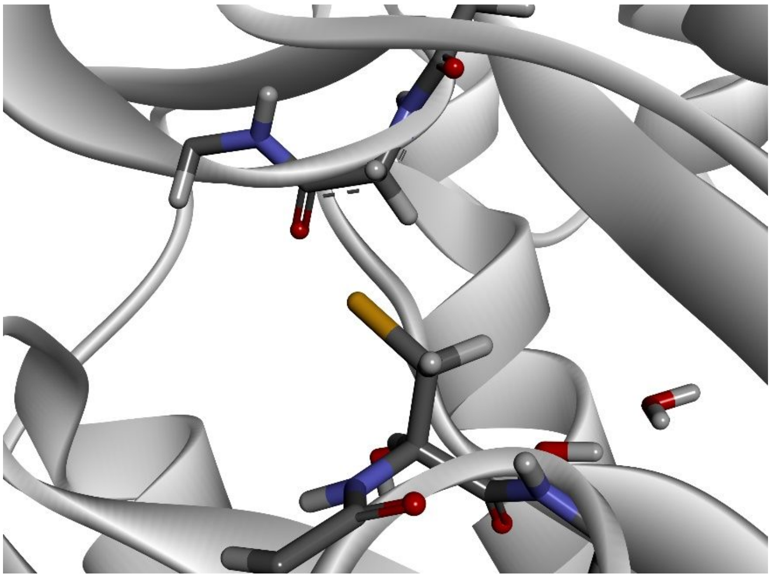

Figure S10. Geometry of stationary points obtained for apoenzyme – *re* and *si* attack.

ES apo H_2O

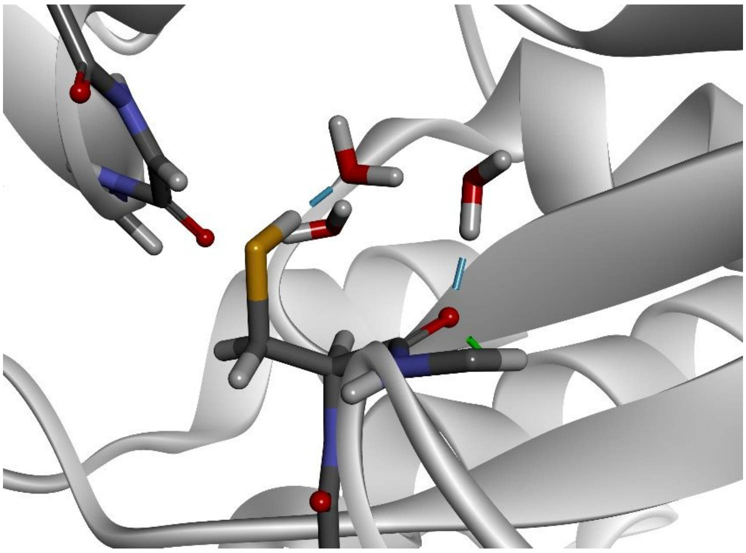

TS apo H_2O

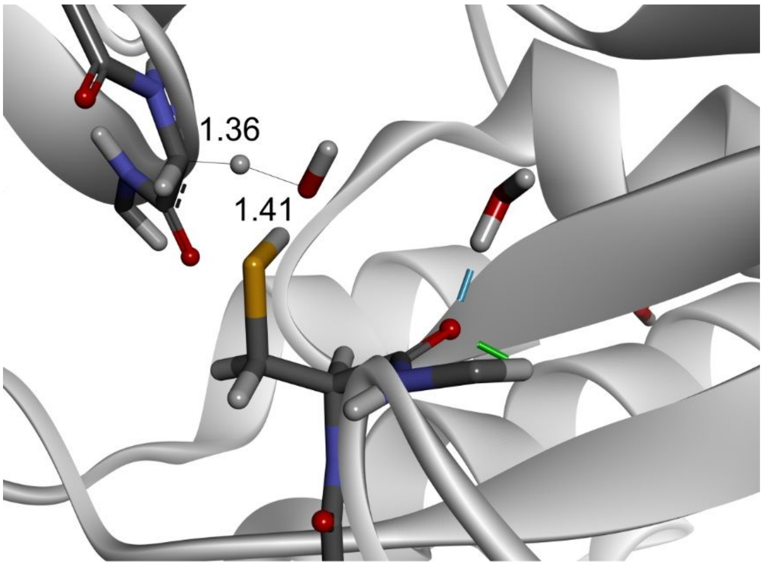

Figure S11. Geometry of stationary points obtained H_2O assisted H transfer assuming a two-step mechanism (1 - Transfer of H atom from H_2O to glycyl radical, 2 - Transfer of H atom from Cys-SH to radical OH)

UHPLC-MS/MS product ion spectra

A)

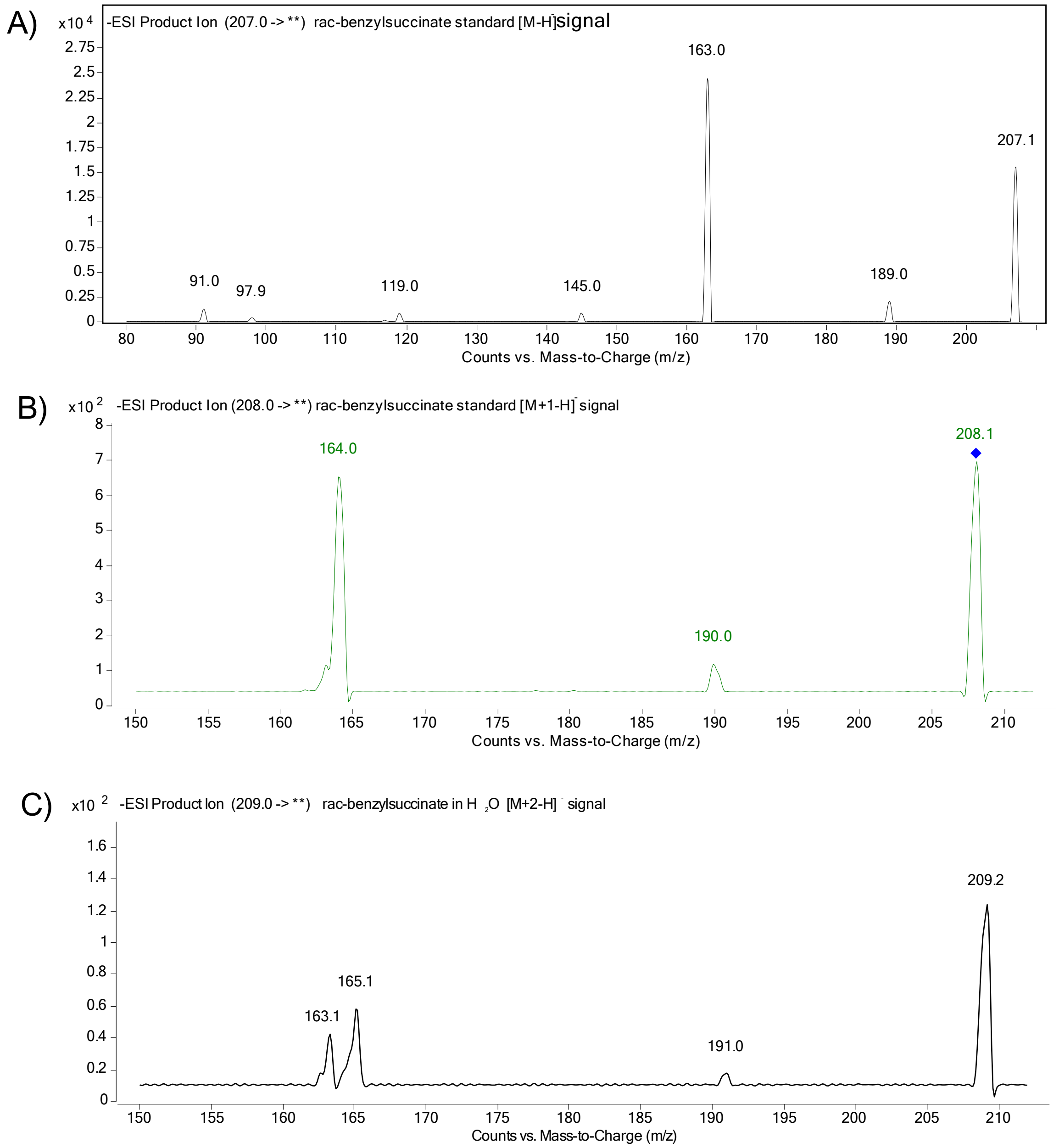

Figure S12. Product ion spectra of *rac*-benzylsuccinate standard for A) quasimolecular ion $[M\text{-}H]^-$, and its isotope signals of B) $[M+1\text{-}H]^-$ and C) $[M+2\text{-}H]^-$.

Product ion fragmentation analysis

The analysis of the MS fragmentation pattern confirmed C3 of benzylsuccinate as the position of both transferred deuterons. The standard fragmentation of benzylsuccinate ion (207 m/z) is the removal of the H_2O group from the carboxylic group and an adjacent carbon atom, yielding a fragmentation ion of $[M-18-H]^-$ at 189 m/z (Fig. S12). In the case of substitution at the C3 atom, we can expect however $[M-19-H]^-$ peak to appear indicating the removal of DOH. In the parent ion of monodeuterated benzylsuccinate (208 m/z, Fig. 8C), the elimination of H_2O predominantly produces a signal at 190 m/z but already a small peak is detected at 189 m/z, indicating the elimination of HOD. However, in the bi-deuterated-product (209 m/z, Fig. 8D) the signal of 190 m/z coming from the elimination of DOH ($[M+d_2\text{-DOH-H}]^-$) is significantly higher than that correlated to H_2O elimination at 191 m/z $[M+d_2\text{-}H_2O\text{-H}]^-$). In the control sample with inactivated BSS, we observed only one signal of 191 m/z associated with the parent ion of 209 m/z, indicating H_2O elimination from isotopomers containing two ^{13}C-atoms (Fig. 8E). We also observed a distinct difference in the fragmentation patterns corresponding to decarboxylation of the quasimolecular ion. In samples where the 209 m/z signal originates from their ^{13}C content (i.e. control experiment with inactive enzyme or *S*-benzylsuccinate), we observed strong fragment ions of 163 and 165 m/z and a small peak at 164 m/z indicating the removal of CO_2 or HCO_2 fragments containing either ^{12}C or ^{13}C carbon atoms. Meanwhile, in the deuterated samples, the 164 or 165 m/z signal was significantly enhanced compared to the 163 m/z signal (for d_1- or d_2-benzylsuccinate, respectively), which proves that the isotope labels were not associated with carboxyl groups.
